# NYX‐2925 induces metabotropic *N*‐methyl‐d‐aspartate receptor (NMDAR) signaling that enhances synaptic NMDAR and α‐amino‐3‐hydroxy‐5‐methyl‐4‐isoxazolepropionic acid receptor

**DOI:** 10.1111/jnc.14845

**Published:** 2019-10-16

**Authors:** M. Scott Bowers, Luisa P. Cacheaux, Srishti U. Sahu, Mary E. Schmidt, Joseph A. Sennello, Katherine Leaderbrand, M. Amin Khan, Roger A. Kroes, Joseph R. Moskal

**Affiliations:** ^1^ Falk Center for Molecular Therapeutics, Biomedical Engineering Northwestern University Evanston Illinois USA; ^2^ Aptinyx, Inc. Evanston Illinois USA

**Keywords:** AMPA, colocalization, LTP, metabotropic, NMDA, trafficking

## Abstract

*N*‐methyl‐d‐aspartate receptors (NMDARs) mediate both physiological and pathophysiological processes, although selective ligands lack broad clinical utility. NMDARs are composed of multiple subunits, but *N*‐methyl‐d‐aspartate receptor subunit 2 (GluN2) is predominately responsible for functional heterogeneity. Specifically, the GluN2A‐ and GluN2B‐containing subtypes are enriched in adult hippocampus and cortex and impact neuronal communication via dynamic trafficking into and out of the synapse. We sought to understand if ((2S, 3R)‐3‐hydroxy‐2‐((R)‐5‐isobutyryl‐1‐oxo‐2,5‐diazaspiro[3,4]octan‐2‐yl) butanamide (NYX‐2925), a novel NMDAR modulator, alters synaptic levels of GluN2A‐ or GluN2B‐containing NMDARs. Low‐picomolar NYX‐2925 increased GluN2B colocalization with the excitatory post‐synaptic marker post‐synaptic density protein 95 (PSD‐95) in rat primary hippocampal neurons within 30 min. Twenty‐four hours following oral administration, 1 mg/kg NYX‐2925 increased GluN2B in PSD‐95‐associated complexes *ex vivo*, and low‐picomolar NYX‐2925 regulated numerous trafficking pathways *in vitro*. Because the NYX‐2925 concentration that increases synaptic GluN2B was markedly below that which enhances long‐term potentiation (mid‐nanomolar), we sought to elucidate the basis of this effect. Although NMDAR‐dependent, NYX‐2925‐mediated colocalization of GluN2B with PSD‐95 occurred independent of ion flux, as colocalization increased in the presence of either the NMDAR channel blocker (5R,10S)‐(–)‐5‐Methyl‐10,11‐dihydro‐5H‐dibenzo[a,d]cyclohepten‐5,10‐imine hydrogen maleate or glycine site antagonist 7‐chlorokynurenic acid. Moreover, while mid‐nanomolar NYX‐2925 concentrations, which do not increase synaptic GluN2B, enhanced calcium transients, functional plasticity was only enhanced by picomolar NYX‐2925. Thus, NYX‐2925 concentrations that increase synaptic GluN2B facilitated the chemical long‐term potentiation induced insertion of synaptic α‐amino‐3‐hydroxy‐5‐methyl‐4‐isoxazolepropionic acid receptor GluA1 subunit levels. Basal (unstimulated by chemical long‐term potentiation) levels of synaptic GluA1 were only increased by mid‐nanomolar NYX‐2925. These data suggest that NYX‐2925 facilitates homeostatic plasticity by initially increasing synaptic GluN2B via metabotropic‐like NMDAR signaling.

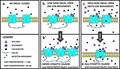

Cover Image for this issue: doi: 10.1111/jnc.14735.

## Graphical abstract

((2S, 3R)‐3‐hydroxy‐2‐((R)‐5‐isobutyryl‐1‐oxo‐2,5‐diazaspiro[3,4]octan‐2‐yl) butanamide (NYX‐2925) is a novel *N*‐methyl‐d‐aspartate (NMDA) modulator that enhances LTP. Here, we sought to determine its mechanism of action. (a) NYX‐2925 engages homeostatic plasticity by exhibiting dose‐dependent mechanism of action at NMDARs (multi‐subunit calcium channels). (b,b′) low picomolar NYX‐2925 increases synaptic post‐synaptic density protein 95 (PSD‐95)‐colocalized *N*‐methyl‐d‐aspartate receptor subunit 2, subtype B (GluN2B)‐containing NMDA receptors (within 30 min). (c,c′) Mid‐nanomolar concentrations flux calcium and decrease synaptic GluN2B after 60 min. In contrast to mid‐nanomolar, the low‐picomolar mechanism appears metabotropic (ion flux independent). Synaptic GluA1 is increased by NYX‐2925 concentrations that mediate ion flux and/or chemical long‐term potentiation, which is further increased by metabotropic‐like NYX‐2925 concentrations. NYX‐2925 is a powerful tool to interrogate both canonical and unconventional modes of signaling through the NMDA receptor.

**Abbreviations**
AAALAC: Association for Assessment and Accreditation of Laboratory Animal Care
aCSF: artificial cerebrospinal fluid
AMPAR: α‐amino‐3‐hydroxy‐5‐methyl‐4‐isoxazolepropionic acid receptor
APV: D‐(‐)‐2‐amino‐5‐phosphonovaleric acid
chemLTP: chemical long‐term potentiation
7CK: 7‐chlorokynurenic acid
DIV: days *in vitro*
EC_50_: concentration at which 50% of the population is activated
FWHM: full width at half maximum
GluA1: amino‐3‐hydroxy‐5‐methyl‐4‐isoxazolepropionic acid receptor subunit 1
GluN1: *N*‐methyl‐d‐aspartate receptor subunit 1
GluN2A: *N*‐methyl‐d‐aspartate receptor subunit 2, subtype A
GluN2B: *N*‐methyl‐d‐aspartate receptor subunit 2, subtype B
LTP: long‐term potentiation
LTD: long‐term depression
MK‐801: (5R,10S)‐(–)‐5‐methyl‐10,11‐dihydro‐5H‐dibenzo[a,d]cyclohepten‐5,10‐imine hydrogen maleate
MS: mass spectral
NBQX: 2,3‐dioxo‐6‐nitro‐1,2,3,4‐tetrahydrobenzo[f]quinoxaline‐7‐sulfonamide disodium salt
NMDAR: *N*‐methyl‐d‐aspartate receptor
NYX‐2925: ((2S, 3R)‐3‐hydroxy‐2‐((R)‐5‐isobutyryl‐1‐oxo‐2,5‐diazaspiro[3,4]octan‐2‐yl) butanamide
PO: per os (by mouth oral administration)
PSD‐95: post‐synaptic density protein 95
RRID: Research Resource Identifier
TTX: tetrodotoxin

*N*‐methyl‐d‐aspartate receptors (NMDARs) are ligand‐gated cation channels thought to play critical roles in brain plasticity and higher cognitive functions (Bliss and Collingridge 1993). NMDAR dysfunction is associated with psychiatric and neurological disorders including depression, neuropathic pain, post‐traumatic stress disorder, and age‐related cognitive decline, among others (Kumar 2015; Aiyer *et al.* 2017; Ghasemi *et al.* 2017; Krystal *et al.* 2017). However, currently available NMDAR ligands exhibit low clinical utility (Chen and Lipton 2006). Accordingly, there is much interest in developing novel NMDAR modulators with therapeutic potential.

The NMDAR is a heterotetrameric complex composed of multiple subunits; the specific combination of these subunits imparts unique biophysical, pharmacological, and signaling properties to channel function (Vicini *et al.* 1998; Cull‐Candy and Leszkiewicz 2004; Traynelis *et al.* 2010; Paoletti 2011). Briefly, all NMDARs contain two obligatory GluN1 subunits (GluN1) and two additional subunits that can be comprised of GluN2 (A through D subtypes) and/or GluN3 (A or B subtype), but the GluN2 subunit is thought to be the major determinant of NMDAR functional heterogeneity (Monyer *et al.* 1992; Ishii *et al.* 1993; Monyer *et al.* 1994; Vicini *et al.* 1998; Cull‐Candy and Leszkiewicz 2004; Gielen *et al.* 2009; Yuan *et al.* 2009; Traynelis *et al.* 2010; Paoletti 2011).

In the adult hippocampus and cortex, NMDAR subunit 2, subtype A (GluN2A) and GluN2B are the predominantly expressed GluN2 subunits (Monyer *et al.* 1994), and NMDARs containing these GluN2 subtypes are heterogeneously partitioned among synaptic, perisynaptic, and extrasynaptic membranes. This partitioning suggests that specific NMDAR composition uniquely impacts neuronal integration of synaptic and extrasynaptic inputs (Groc *et al.* 2009). Previous work has found that the GluN2A and GluN2B subtypes are dynamically trafficked into and out of the synaptic membrane by NMDAR agonists (Barria and Malinow 2002) and co‐agonists (Nong *et al.* 2003; Ferreira *et al.* 2017). Thus, NMDARs (particularly those containing the GluN2B subtype) are highly mobile and can exchange between synaptic and extrasynaptic sites (Tovar and Westbrook 2002; Groc *et al.* 2006; Ferreira *et al.* 2017).

Here, we sought to understand if the NMDAR modulator, ((2S, 3R)‐3‐hydroxy‐2‐((R)‐5‐isobutyryl‐1‐oxo‐2,5‐diazaspiro[3,4]octan‐2‐yl) butanamide (NYX‐2925), regulates synaptic levels of GluN2A and GluN2B. NYX‐2925 is an orally bioavailable non‐peptide mimetic of GLYX‐13, which is also known as Rapastinel. In rat models, both NYX‐2925 (Khan *et al.* 2018) and GLYX‐13 (Moskal *et al.* 2017) are cognitive enhancers that facilitate synaptic plasticity. In brief, we found that NYX‐2925 exhibits a unique biphasic dose response. Mid‐nanomolar concentrations mediate calcium transients and long‐term potentiation (LTP) (Khan *et al.* 2018), whereas picomolar concentrations engage hippocampal homeostatic plasticity by augmenting synaptic levels of GluN2B via a metabotropic‐like mechanism of the NMDAR. Next, we found that NYX‐2925 pretreatment enhanced activity‐dependent synaptic insertion of α‐amino‐3‐hydroxy‐5‐methyl‐4‐isoxazolepropionic acid receptors (AMPARs). Given the NYX‐2925 dose–response profile and increasing appreciation of non‐ionotropic modes of NMDAR signal transduction (Dore *et al.* 2017), this study demonstrates that NYX‐2925 is a powerful tool to probe both canonical and novel modes of NMDAR signaling.

## Methods

### Reagents

All reagents were obtained in the highest quality possible from standard vendors. NYX‐2925 [((2S, 3R)‐3‐hydroxy‐2‐((R)‐5‐isobutyryl‐1‐oxo‐2,5‐diazaspiro[3.4]octan‐2‐yl) butanamide] was synthesized by Sai Life Sciences (Telangana, India). D‐(‐)‐2‐amino‐5‐phosphonovaleric acid (APV; PubChem ID: 135342), 7‐chlorokynurenic acid (7CK; PubChem ID: 1884), (5R,10S)‐(–)‐5‐Methyl‐10,11‐dihydro‐5H‐dibenzo[a,d]cyclohepten‐5,10‐imine hydrogen maleate (MK‐801; PubChem ID: 6420042), tetrodotoxin (TTX; PubChem ID: 16759596), strychnine (PubChem ID: 16219987), and bicuculline (PubChem ID: 6852386) were obtained from Tocris (Minneapolis, MN, USA). 2,3‐Dioxo‐6‐nitro‐1,2,3,4‐tetrahydrobenzo[f]quinoxaline‐7‐sulfonamide disodium salt (PubChem ID: 3272523) and nimodipine (PubChem ID: 4497), and NMDA (Cat #: ab120052) were obtained from Abcam (Cambridge, MA, USA). Unless otherwise noted, cell culture reagents were obtained from Fisher (Gibco, Waltham, MA, USA; RRID: SCR_008452).

### Animal housing and treatment

Animal use was approved by the Northwestern University Institutional Animal Care and Use Committee [OLAW assurance: D16‐00182 (A3283‐01) obtained expiration 5/31/22; Association for Assessment and Accreditation of Laboratory Animal Care accreditation 10/9/85]. After delivery, timed dams (Envigo, Indianapolis, IN, USA) were allowed to acclimate in an Association for Assessment and Accreditation of Laboratory Animal Care‐accredited vivarium for at least 24 h before embryos were harvested. Food and water were provided *ad libitum* and lights were on a 12‐h cycle (lights on 06:00). For *in vivo* studies, P60 male Sprague Dawley rats (Envigo) were housed 3 per cage for 1 week in the vivarium prior to experimentation. Next, rats (*n* = 3 animals per treatment group; no animals were excluded) received vehicle (1 mL/kg, 0.5% carboxy methyl cellulose in water, P.O.) or NYX‐2925 (1 mg/kg, 0.5% carboxymethylcellulose in water, P.O.). Animals were not anesthetized during drug treatment. Twenty‐four hours after treatment, the prefrontal cortex was isolated on ice and snap frozen. All animals were killed by rapid decapitation. Power analysis was not performed, no data were excluded (no exclusion criteria were pre‐determined), and no animals died during experimentation.

### Primary culture

Primary hippocampal cultures were prepared as previously described (Nault and De Koninck 2010). Briefly, hippocampi were extracted from embryonic day 18 Sprague‐Dawley rat pups (Charles River Laboratories, Wilmington, MA, USA; RRID:RGD_737891) and brains from a given litter pooled and placed into cold Hibernate E. Tissue was washed and chopped with a scalpel prior to enzymatic digestion (10 min, Papain, BrainBits, Springfield, IL, USA) at 37°C and subsequent washing with plating media (MEM Cat #: 11090081, supplemented with 10% fetal bovine serum Cat #: 26400044 from Hyclone, Logan, UT, USA; 2 mM Glutamax^®^ Cat #: 35050061; 1 mM sodium pyruvate Cat #: 111360070; and 10 μg/mL penicillin/ 10 μg/mL streptomycin Cat #: 15‐140‐122). Next, cells were triturated with a polished pipette, strained (100 μm), and dropped onto 12 mm glass poly‐d‐lysine coverslips (neuVitro, Vancouver, WA, USA, Cat #: CC‐12‐pdl) at 3.5 × 10^5^ cells per mL. After 90 min, cells were flooded with un‐supplemented Neurobasal^®^ (Cat #: 21‐103‐049) and plating media applied. Four hours later, 50% of the media was exchanged with Neurobasal^®^ that was supplemented with B27, Glutamax^®^, and penicillin/streptomycin. Three days later, cells were treated with 3 μM cytosine arabinoside (AraC; Sigma‐Aldrich, St. Louis, MO, USA, Cat #: C1768) for 24 h and 50% of the media was replaced with fresh, supplemented Neurobasal^®^. Thereafter, cells were fed twice per week by exchanging 50% of the media with supplemented Neurobasal^®^ until cultures were used at 14–15 days *in vitro*.

### NYX‐2925 treatment

All solutions were prepared and brought to 37°C on the day of the experiment. Treatment groups consisted of coverslips (prepared as described above) from multiple plates, and all experiments were run in at least duplicate from independent cell culture preparations. Cells were washed with artificial cerebrospinal fluid (aCSF; in mM: 119 NaCl, 30 glucose; 10 HEPES; 5 KCl; 2 CaCl_2_, 2 MgCl_2_, pH 7.4) and allowed to recover in aCSF at 37°C for 30 min. Next, vehicle or glutamate (final concentration 50 μM) and/or NYX‐2925 (final concentration 0.1 pM–30 nM) were co‐applied for 30 or 60 min. The rationale for quantifying drug effect at these time points was based upon the findings that NYX‐2925 facilitated LTP when bath applied to rat hippocampal slices for 40 min and that the maximum concentration of NYX‐2925 in the rat cerebral spinal fluid occurs 60 min after oral dosing (Khan *et al.* 2018). Cells were washed (cold PBS) and then processed for immunocytochemistry, harvested in RIPA with protease and phosphatase inhibitors (1 : 100, Sigma‐Aldrich). Blockers of the NMDAR glutamate site (APV), glycine site (7CK), or channel (MK‐801) were included in both the recovery and drug‐treatment buffer, when indicted below. Because MK‐801 is an activity‐dependent channel blocker, MK‐801 was both pre‐incubated (30 min) and co‐applied with NYX‐2925. Application of MK‐801 in this manner blocks any NMDARs that may be activated by ligand or spontaneous activity of primary hippocampal cultures (Siebler *et al.* 1993). 7CK was similarly used with both a pre‐incubation and co‐application.

### Immunocytochemistry of GluN2‐containing NMDARs and PSD‐95

After washing with cold PBS, cells were fixed on ice with cold 4% p‐formaldehyde (EMS, Hatfield, PA, USA, Cat #: 50‐980‐487) in 4% sucrose/PBS for 7 min. After washing, cells were blocked in 1% normal goat serum (MP Biomedicals, Santa Ana, CA, USA, Cat #: ICN642921) at 4°C and then incubated with mouse primary antisera directed against the extracellular domain of GluN2A (1 : 800, Biolegend, San Diego, CA, USA; RRID: AB_2564953) or GluN2B (1 : 1000, Biolegend; RRID: AB_2564823). After washing, secondary antibody incubation (1 : 200, goat anti‐mouse AF594, Probes, Waltham, MA, USA, RRID: AB_2534091), subsequent wash, and post‐fix (2% p‐formaldehyde, 5 min, 4°C), cells were permeabilized (0.25% Triton X‐100^®^ Cat #: BP151), and intracellular epitopes blocked (1% normal goat serum). Next, endogenous biotin was blocked (Vector Labs, Burlingame, CA, USA, Cat #: NC9406552) prior to incubation with mouse biotinylated primary directed against PSD‐95 (1 : 1000, SYSY, Goettingen, Germany; RRID: AB_10804286) and chicken primary antisera directed against microtubule associated protein 2 (MAP2; 1 : 5000, Abcam; RRID: AB_2138153). Primary antisera were detected with NeutrAvidin Dylight^®^ 488 (1 : 200, Fisher Scientific, Waltham, MA, USA; Cat #: PI22832) or goat anti‐chicken AF647 (1 : 3000, Probes; RRID: AB_2535866) prior to mounting in Prolong Gold (Probes). Antisera were centrifuged (10 000 *g*, 5 min, 4°C) immediately prior to use.

### Immunoblotting

Neurons were collected in RIPA buffer with protease inhibitors (1 : 100, Fisher; Cat #: 78444), sonicated, and centrifuged (20 min, 14 000 *g*, 4°C). Protein in supernatant was quantified (Pierce 660 nm assay, Thermo Scientific, Waltham, MA, USA; Cat #: PI22663). Thirty‐five micrograms of protein were run on a 4–20% gradient polyacrylamide gel and transferred onto a 0.22 µm nitrocellulose membrane. Blots were blocked in 5% non‐fat dry milk (Bio‐Rad, Hercules, CA, USA, Cat #: 1706404) and incubated with primary antibody as follows: GluN2A (1 : 1000, Cell Signaling, Beverly, MA, USA, overnight, 4°C, RRID: AB_2112295), GluN2B (1 : 1000, Cell Signaling, overnight, 4°C, RRID: AB‐1264223), PSD‐95 (1 : 5000, Millipore, Burlington, MA, USA, overnight, 4°C, RRID: AB_10807979) or β‐actin HRP (1 : 5000, Cell Signaling, 1 h, (20℃), RRID: AB_1903890), followed by host‐specific secondary antibody for 1 h at 20℃ (1 : 2000 of goat anti‐rabbit HRP (Cell Signaling, RRID: AB_2099233) or goat anti‐mouse Alexa Fluor^®^ 647 (Probes, RRID: AB_2535804). Blots were imaged (Bio‐Rad Chemidoc) and band intensity was analyzed using Image Lab 6.0.1 (Bio‐Rad).

### Proteomic analysis of rat hippocampal cultures

#### Sample preparation

Hippocampal cultures, prepared as above, were treated with either 1 pM or 30 nM NYX‐2925 for 30 min, also as described above (*n* = 4 independent cell culture preparations). Treated cultures were washed in cold PBS and frozen at −80°C until analysis. Subsequent sample handling, mass spectrometry, and data analysis were performed by MSBioworks LLC (Ann Arbor, MI, USA). Cells were lysed in 500 μL urea buffer (8 μM urea, 50 mM Tris HCl, 150 mM NaCl, 1X Roche (Indianapolis, IN, USA) Complete Proteinase Inhibitor (Sigma‐Aldrich, Cat #: 11836145001, pH 8.0). Samples were incubated at 20℃ for 1 h, clarified by centrifugation, and quantified by Qubit fluorometry (Life Technologies, Rockville, MD, USA). A 50 μg aliquot of each sample was digested in solution using the following protocol: sample was diluted in 25 mM ammonium bicarbonate (Cat #: A643), reduced with 10 mM dithiothreitol (Cat #: AAJ1539706) at 60°C, alkylated using 50 mM iodoacetamide (Cat #: AC122270050) at 20℃, digested with 2.5 μg trypsin (Promega, Madison, WI, USA, Cat #: V5280) at 37°C for 18 h followed by quenching with formic acid. Peptides were cleaned using solid‐phase extraction using the Empore C18 plate (3M, St. Paul, MN, USA, Cat #: 14‐378‐57).

#### Mass spectrometry

Peptides (2 μg per sample) were analyzed by nano LC/MS/MS with a NanoAcquity HPLC system (Waters) interfaced to a Fusion Lumos mass spectrometer (Thermo Fisher, Burlington, MA, USA). Peptides were loaded on a trapping column and eluted over a 75‐μm analytical column at 350 nL/min; both columns were packed with Luna C18 resin (Phenomenex, Torrance, CA, USA, Cat #: 00G‐4252‐E0). Each sample was separated over a 4‐h gradient. The mass spectrometer was operated in data‐dependent mode, with MS and MS/MS performed in the Orbitrap at 60 000 full width at half maximum (FWHM) resolution and 15 000 FWHM resolution, respectively. The instrument was run with a 3‐s cycle for MS and MS/MS.

#### Data processing

Data were processed through the MaxQuant software v1.6.0.16 (http://www.maxquant.org), normalized using the LFQ algorithm, peptide IDs searched using Andromeda (Cox *et al.* 2011) with peptide and protein false discovery rate (FDR) < 0.01, and analyzed using Perseus v1.5.5.3 (http://www.coxdocs.org). Statistically significant differences in protein content between drug‐treated and vehicle groups were assessed by Student’s *t*‐test. The level of statistical significance was set at *p* < 0.05.

#### Ontological analyses

Ingenuity^®^ Pathway Analysis (IPA^®^; Qiagen, Germantown, MD, USA) was used to identify overrepresented canonical pathways associated with significantly differentially expressed proteins (*p* < 0.05) identified in the proteomic data sets. The IPA algorithm interrogates a published, curated database of protein interrelationships (Krämer *et al.* 2014) to identify coordinated regulation of biological pathways associated with drug treatment. Multiple hypothesis correction based on the Benjamini–Hochberg approach at 1% FDR threshold was implemented. Statistical significance of pathway enrichment was determined by Fisher’s exact test (*p* < 0.05).

### Proteomic analysis of rat prefrontal cortex PSD‐95 co‐immunoprecipitates

#### PSD‐95 co‐immunoprecipitation

Tissues from rat prefrontal cortex (*n* = 3 per treatment group) isolated 24 h following drug treatment were lysed by sonication in RIPA buffer with protease inhibitors (1 : 100, Sigma). Following centrifugation (10 min, 14 000 *g*, 4°C), protein in supernatant was quantified (BCA, Thermo Scientific). Ten micrograms of PSD‐95 antibody (Millipore) was incubated with 6 mg of magnetic beads (Dynabeads Protein G, Thermo Scientific, Burlington, MA, USA, 2 h, 4°C) and crosslinked [5 mM bis‐sulfosuccinicimidyl suberate (BS3), Thermo Fisher, Burlington, MA, USA] for 30 min prior to quenching (1 M Tris–HCl, pH 7.5, 15 min) and overnight incubation with 1 mg of rat prefrontal cortex protein. Antibody‐bound protein was eluted by boiling beads (100°C, 5 min), and eluent was analyzed by mass‐spectrometry.

#### MS sample preparation

Each sample was separated on a 10% Bis‐Tris Novex^®^ mini‐gel (Life Technologies) using the 2‐(N‐morpholino)ethanesulfonic acid buffer system. The gel was stained with Coomassie and each lane excised into ten equally sized segments. Gel pieces were robotically processed (ProGest, DigiLab, Hopkinton, MA, USA) with the following protocol: (i) washed with 25 mM ammonium bicarbonate followed by acetonitrile, (ii) reduced with 10 mM dithiothreitol at 60°C followed by alkylation with 50 mM iodoacetamide at 20℃, (ii) digested with trypsin (Promega) at 37°C for 4 h and quenched with formic acid. The supernatant was analyzed directly without further processing.

#### Mass spectrometry

Each gel digest was analyzed by nano LC/MS/MS with a NanoAcquity^®^ HPLC system (Waters, Milford, MA, USA) interfaced to a Q Exactive^®^ (Thermo Fisher). Peptides were loaded on a trapping column and eluted over a 75 μm analytical column at 350 nL/min; both columns were packed with Jupiter Proteo^®^ resin (Phenomenex). The mass spectrometer was operated in data‐dependent mode, with MS and MS/MS performed in the Orbitrap (Thermo Fisher) at 70 000 FWHM and 17 500 FWHM resolution, respectively. The fifteen most abundant ions were selected for MS/MS.

#### Data processing

Data were searched using Mascot (Matrix Scientific, Boston, MA, USA) using 10 ppm peptide mass tolerance, 0.02 Da fragment mass tolerance with a maximum of two missed cleavages. Mascot DAT files were parsed into Scaffold (Proteome Software, Portland, OR, USA) for validation, filtering, and to create a non‐redundant list per sample. Data were filtered at 1% protein and peptide level FDR requiring at least two unique peptides per protein.

### Calcium transients

Calcium imaging was performed on primary hippocampal (or cortical for Supporting Information) neurons at days in vitro 20–22. Cells were loaded with 2 μM Fluo‐4‐AM (Thermo Fisher, Cat #: F14202) dissolved in pluronic F‐127 (Cat #: P3000MP) and 20% anhydrous DMSO (Molecular Probes, Cat # D12345) for 20 min at 20℃ in buffer (in mM: 145 NaCl, 5.4 KCl, 1.8 CaCl_2_, 11 glucose, 10 HEPES, pH 7.4). For dye loading, 50 μM APV was also added to the buffer. After dye loading, cells were washed three times with buffer containing APV and left at 20℃ for 15 min before imaging to complete deesterification of the dye. The cells were then washed with extracellular buffer without APV and transferred to a microscope chamber. Extracellular buffer containing 500 nM TTX, 10 μM 2,3‐Dioxo‐6‐nitro‐1,2,3,4‐tetrahydrobenzo[f]quinoxaline‐7‐sulfonamide disodium salt (2 μM for cortical cultures), and 10 μM nimodipine (0 M for cortical) was perfused onto the cells for 3–4 min to wash off the APV. Fluo‐4‐AM fluorescence in the cells was detected with an Eclipse Ti2 fluorescent microscope (Nikon, Tokyo, Japan), SOLA SE II 365 Light Engine (Lumencor, Beaverton, OR, USA), and DS‐Fi3 camera (Nikon) at 20× magnification. The experiments were performed at 20℃, and images were acquired at 1 frame/2 s. After acquisition of a 1.5 min baseline, 4 μM NMDA was washed onto the cells for 1 min followed by a 2‐min washout. NYX‐2925 (either 1 pM or 30 nM) plus 4 μM NMDA was then washed onto the cells for 1 min followed by a 2‐min washout. 10 μM NMDA plus 3 μM D‐serine was washed onto the cells at the end to elicit a ‘max’ response. For data analysis, relative fluorescence intensity (Δ*F*/*F*_0_) was calculated. The maximum Δ*F*/*F*_0_ values for the initial NMDA response as well as for NYX‐2925 + NMDA were used to calculate % enhancement values. Cells whose initial NMDA response was larger than 40% of the ‘max’ response were excluded.

### Chemical induction of LTP (chemLTP)

ChemLTP was conducted as previously described (Lu *et al.* 2001). Following vehicle or NYX‐2925 treatment (30 min), cells were washed and placed into chemLTP buffer (in mM: 140 NaCl, 33 glucose, 25 HEPES, and 2 CaCl_2_; in μM: 200 glycine, 25 bicuculline, 1 TTX, and 1 strychnine) for 3 min at 37°C prior to being placed in recovery buffer for 20 min (in mM: 140 NaCl, 33 glucose, 25 HEPES, and 2 CaCl_2_; in μM: 25 bicuculline, 1 TTX, and 1 strychnine) at 37°C. The chemLTP vehicle cohort was placed into recovery buffer twice instead of chemLTP buffer followed by recovery buffer. Next, cells were washed (cold PBS) and fixed on ice (4% p‐formaldehyde, 4% sucrose, in PBS) for 13 min prior to blocking (10% donkey serum, Sigma‐Aldrich, Cat #: D9663) and incubation with rabbit primary antisera for GluA1 (1 : 10, 1 h, 4°C, Millipore, RRID: AB‐564636) as described (Ohashi *et al.* 2016), and cy3‐labelled secondary (Jackson ImmunoResearch, West Grove, PA, USA, RRID: AB_2307443). Immunocytochemistry for PSD‐95 and MAP2 proceeded as described above.

### Confocal microscopy and image analysis

Images were acquired with a Yokogawa CSU‐X1 spinning disk (Tokyo, Japan) by a blinded experimenter. All images were taken with a Plan Apo 60X oil objective (1.42 numerical aperture) mounted on a Ti2 confocal microscope (Nikon) equipped with an FRAPPA camera (Andor, Belfast, UK) and NIS‐Elements AR 4.51.01 (Nikon). Image stacks were acquired at Nyquist (generally 50–60 individual planes), each 512 × 512 pixels in size (12 bit). Refractive index was 1.515 and pinhole was 45.98 μm. Channel acquisition order was far‐red, red, green, and then blue. Laser power, exposure time, and averaging were optimized to avoid saturation and parameters were held constant across experiments. No more than four images were acquired from a single coverslip across 4 coverslips in at least duplicate from independent cell culture preparations. Images were processed by NIS Elements (Nikon) and analyzed with Volocity 6.1.1 (Quorum, Puslinch, ON, Canada). A 1.5 lower limit standard deviation threshold was applied to images, and based upon ultrastructural measurements of the post‐synaptic density (Sheng and Hoogenraad 2007), immunolabeled puncta were identified GluN2‐containing NMDAR and GluA1‐containing AMPARs (Receptor: 10 μM radius, 1 μM^3^ minimum object size; PSD‐95: 10 μM radius, 0.5 μM^3^ minimum object size). Colocalization was measured as the mean intensity of GluN2 subtypes or GluA1 subunit puncta within PSD‐95 puncta. A field of view contributed one n to the analysis. Thus, mean colocalization of 14–28 cells (from at least two independent cell culture preparations) were analyzed per treatment.

### Statistical analysis

**Figure 1:**
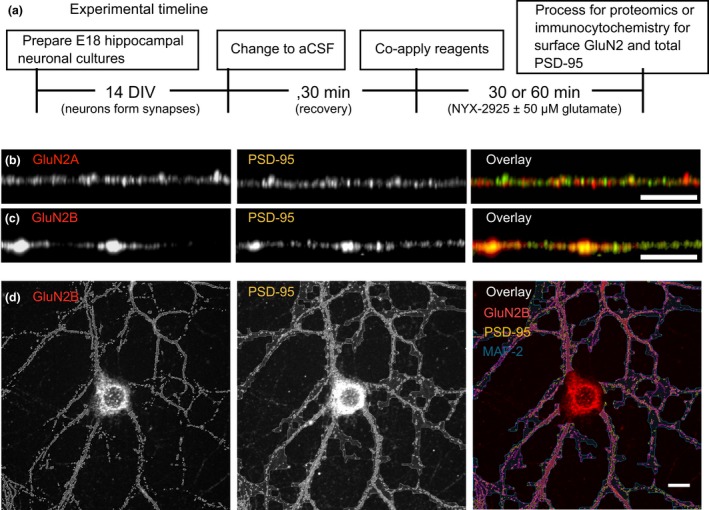
Experimental design for analysis of colocalization of *N*‐methyl‐d‐aspartate receptor subunit 2 (GluN2)‐contianing NMDARs with post‐synaptic density protein 95 (PSD‐95). (a) Primary hippocampal neurons derived from E18 pups were cultured for 14 days prior to exchanging media with artificial cerebrospinal fluid (aCSF). Cells were allowed to recover in aCSF for 30 min at 37°C. Next, ((2S, 3R)‐3‐hydroxy‐2‐((R)‐5‐isobutyryl‐1‐oxo‐2,5‐diazaspiro[3,4]octan‐2‐yl) butanamide (NYX‐2925), with or without 50 μM glutamate, was co‐applied for either 30 or 60 min prior to processing cells for immunocytochemistry or proteomics. (b) Representative dendrites immunolabeled of GluN2A and PSD‐95. (c) Representative dendrites immunolabeled for GluN2B and PSD‐95. (d) Representative immunolabeling of GluN2B and PSD‐95 with computer‐identified immunopositive puncta delineated. Microtubule associated protein 2 (MAP2) was used as a marker for neurons and general index of cell health. Scale bar = 10 μm.

Outliers were determined as lying greater than two standard deviations from the mean for each treatment group. Exclusions include Fig. 1f (*n* = 1 from 1 nM), Fig. 3a (*n* = 2 from pM, *n* = 1 pM + MK801), Fig. 3c (*n* = 1 from 1 nM. After determining normal distribution of the data (K‐S Normality Test), data were analyzed by one‐way anova or paired Student’s *t*‐test, as indicated, at 95% confidence (Statview 5.0, Berkeley, CA, USA). Where appropriate, significant main effects were investigated by *post hoc* analysis, as indicated in the figure legends. All data are presented as mean ± the standard error of the mean with whiskers indicating data spread.

## Results

### NYX‐2925 increases synaptic GluN2B *in vitro*

Rat primary hippocampal neurons were cultured for 14 days prior to bath application of NYX‐2925 with or without 50 μM glutamate for 30 or 60 min (Fig. 1a). Dose selection was based on the previous finding that picomolar NYX‐2925 in the presence of 50 μM glutamate selectively potentiates [^3^H]MK‐801 binding to GluN2B‐containing receptors, whereas nanomolar NYX‐2925 does so in all GluN2 subtypes, including GluN2A (Khan *et al.* 2018). Following treatment, cells were washed, fixed, and immunolabeled for surface GluN2A or GluN2B and intracellular PSD‐95 to determine the effects of NYX‐2925 treatment on synaptic localization of GluN2‐containing NMDARs. Representative immunolabeling and specific fluorophore detection of GluN2A, GluN2B, and PSD‐95 is indicated (Fig. 1b–d). NMDAR colocalization with PSD‐95 is used as a proxy for synaptically localized receptor because it is generally thought that the NMDAR is retained in the synaptic membrane via binding of the C‐terminal domain to PDZ domain‐containing proteins, including PSD‐95 (Prybylowski *et al.* 2005; Al‐Hallaq *et al.* 2007; Bard *et al.* 2010).

**Figure 2:**
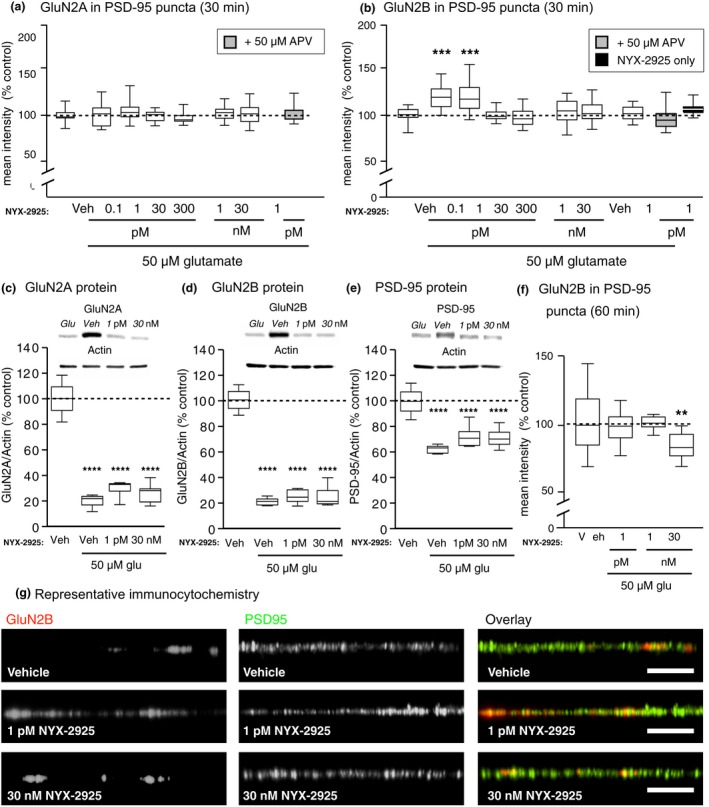
Quantification of colocalization of *N*‐methyl‐d‐aspartate receptor subunit 2 (GluN2) with post‐synaptic density protein 95 (PSD‐95). (a) Colocalization of GluN2A with PSD‐95 was unaffected by ((2S, 3R)‐3‐hydroxy‐2‐((R)‐5‐isobutyryl‐1‐oxo‐2,5‐diazaspiro[3,4]octan‐2‐yl) butanamide (NYX‐2925) treatment in the presence 50 μM glutamate. *n* = 14 cells from two independent cell culture preparations (eight coverslips). (b) After incubating primary hippocampal neurons with various NYX‐2925 concentrations in the presence of 50 μM glutamate, a significant increase in colocalization of GluN2B with PSD‐95 was seen for 0.1 and 1 picomolar concentrations only. This NYX‐2925‐mediated increase in colocalization was blocked by the NMDAR glutamate site antagonist D‐(‐)‐2‐amino‐5‐phosphonovaleric acid (APV) (grey). The antagonist APV was applied via both a 30 min pre‐treatment as well as during the 30 min co‐incubation with NYX‐2925 and glutamate. No change in colocalization was observed if neurons were treated for 30 min with either 1 picomolar NYX‐2925 alone or 50 μM glutamate alone. *n* = 14 cells from two independent cell culture preparations (eight coverslips). Whole‐cell immunocontent of (c) GluN2A, (d) GluN2B, and (e) PSD‐95 were decreased after 30 min incubation of NYX‐2925 in the presence of 50 μM glutamate, but no difference was seen between either NYX‐2925 concentration in the presence of 50 μM glutamate compared to glutamate alone. *n* = 5–9 independent cell culture preparations (whole‐cell lysates). (f) No change in colocalization of GluN2B with PSD‐95 was observed after incubating neurons with picomolar NYX‐2925 concentrations in the presence of 50 μM glutamate for 60 min, but colocalization was decreased by continuous exposure to 30 nM NYX‐2925 with 50 μM glutamate. *n* = 14 cells from two independent cell culture preparations (eight coverslips). (g) Representative dendrites immunolabeled for GluN2B and PSD‐95. Scale bar = 10 μm. Veh: vehicle; Glu: glutamate. Data represent mean ± SEM ± data spread; the line within each box represents the average, the box itself represents the SEM, and the whiskers indicate the spread of the data. **p < 0.01, ***p < 0.001 Tukey *post‐hoc* analysis, *n* = 14 cells per treatment; ****p < 0.0001 Dunnett’s *post hoc* analysis.

Using this approach, the effect of NYX‐2925 on synaptic GluN2A and GluN2B levels was studied. Application of NYX‐2925 (0.1 pM–30 nM) in the presence of glutamate for 30 min did not increase colocalization of GluN2A with PSD‐95 in primary neurons (Fig. 2a). Next, an expanded dose‐response curve was conducted for the GluN2B subtype. After 30 min, synaptic GluN2B increased only at low picomolar NYX‐2925 concentrations in the presence of glutamate [*F*(9, 130) = 7.019, *p* = 0.0001, *n* = 14 cells per group; Fig. 2b]. The increase in colocalization seen after NYX‐2925 treatment is NMDAR‐dependent because colocalization did not increase in the presence of the NMDAR glutamate site antagonist APV (compared to vehicle, *p* > 0.05; compared to NYX‐2925, *p* < 0.05; Fig. 2b). Moreover, *post hoc* analysis showed that neither 1 picomolar NYX‐2925 nor glutamate alone were sufficient to increase synaptic GluN2B (both compared to vehicle, *p* > 0.05; Fig. 2b). Whole‐cell protein content of GluN2A, GluN2B, and PSD‐95 were decreased by glutamate, but not further altered by either 1 picomolar or 30 nanomolar NYX‐2925 [Fig. 2c–e; GluN2A: *F*(3, 26) = 1.46, *p* < 0.0001; GluN2B: *F*(3, 26) = 0.94, *p* < 0.0001; PSD‐95: *F*(3, 26) = 34.52, *p* < 0.001]. The increase in synaptic GluN2B levels mediated by low picomolar NYX‐2925 appears to be transient *in vitro*, as colocalization returned to baseline after continuous drug treatment at the same concentration for 60 min [*F*(1, 26) = 1.01, *p* = 0.32, *n* = 14 cells per group; Fig. 2f]. Continuous exposure to higher NYX‐2925 concentrations (30 nM) for 60 min significantly reduced colocalization of GluN2B with PSD‐95 [*F*(3, 52) = 5.66, *p* = 0.002, *n* = 14 cells per group; Fig. 2f]. In contrast to the transient effects induced by continuous exposure to low NYX‐2925 concentrations, increased GluN2B levels were seen in PSD‐95 coimmunoprecipitates of the rat prefrontal cortex 24 h after dosing (1 mg/kg, p.o.; Supporting Information; the relationship of this dose with the concentrations studied *in vitro* is discussed below). Lastly, representative immunolabeling is indicated (Fig. 2g). Together, these data indicate that picomolar concentrations of NYX‐2925 function with glutamate via the NMDAR to increase synaptic localization of GluN2B.

### NYX‐2925 increases synaptic GluN2B independent of ion flux through NMDARs

**Figure 3:**
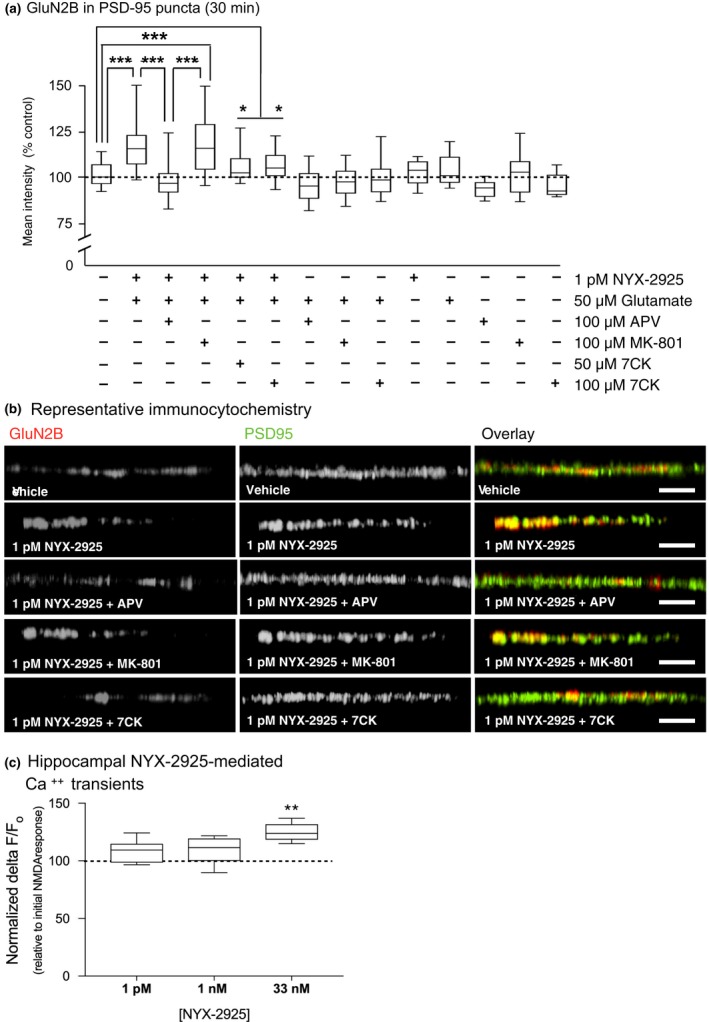
((2S, 3R)‐3‐hydroxy‐2‐((R)‐5‐isobutyryl‐1‐oxo‐2,5‐diazaspiro[3,4]octan‐2‐yl) butanamide (NYX‐2925) mediates *N*‐methyl‐d‐aspartate receptor subunit 2, subtype B (GluN2B) colocalization with post‐synaptic density protein 95 (PSD‐95) via a metabotropic‐like mechanism. Neurons were treated as before with NYX‐2925 and glutamate and the effect of *N*‐methyl‐d‐aspartate receptor (NMDAR) blockers on GluN2B colocalization with PSD‐95 quantified. (a) Co‐application of 1 picomolar NYX‐2925 with 50 μM glutamate increased colocalization of GluN2B with PSD‐95, and this effect was blocked by the glutamate site antagonist D‐(‐)‐2‐amino‐5‐phosphonovaleric acid. In contrast, significantly increased colocalization was observed when either the channel blocker MK‐801 or the glycine site antagonist 7CK were applied. As before, antagonists were both applied before and during NYX‐2925/glutamate incubation. Colocalization was not different from control conditions following application of these antagonists with glutamate alone (in the absence of NYX‐2925) or with NYX‐2925 alone. *n* = 14–28 cells per treatment from two independent cell culture preparations (eight coverslips). (b) Representative dendrites immunolabeled for GluN2B and PSD‐95. (c) Effects of NYX‐2925 on calcium transients following NMDAR activation. Primary hippocampal neurons were loaded with Fluo‐4‐AM, and calcium imaging performed. After a baseline measurement, 4 μM NMDA was washed onto the cells for 1 min followed by a 2‐min washout. NYX‐2925 + NMDA was then washed onto the cells to determine if NYX‐2925 enhanced NMDA receptor mediated calcium transients. Two independent culture preparations were used (*n* = 6 cover slips). Scale bar = 10 μm. Data represent mean ± SEM ± data spread; the line within each box represents the average, the box itself represents the SEM, and the whiskers indicate the spread of the data. ****p < 0.0001 Student’s *t*‐test; ***p < 0.001 Tukey *post hoc* analysis; **p < 0.01 Tukey *post hoc* analysis, *p < 0.05 Fisher *post hoc* analysis.

Given that nanomolar NYX‐2925 concentrations facilitate synaptic plasticity *ex vivo* (Khan *et al.* 2018), it is intriguing that picomolar, but not nanomolar concentrations increased synaptic GluN2B. Accordingly, we sought to better understand how NYX‐2925 acts on GluN2B‐containing NMDARs. As before, primary hippocampal neurons were co‐treated with 1 picomolar NYX‐2925 and 50 μM glutamate for 30 min, and colocalization of GluN2B with PSD‐95 was measured. A one‐way ANOVA found a main effect of treatment [*F*(13, 238) = 7.72, *p* = 0.0001, *n* = 14–28 cells], and *post‐hoc* analysis showed increased colocalization (*p* < 0.05) that was blocked by APV (compared to vehicle, *p* > 0.05; compared to NYX‐2925 + glutamate, *p* < 0.05; Fig. 3a). However, the NYX‐2925‐mediated increase in colocalization was not prevented by either the NMDAR channel blocker MK‐801 (compared to vehicle, *p* < 0.05; compared to NYX‐2925 + glutamate; *p* > 0.05) or the glycine site antagonist 7CK (compared to vehicle, *p* < 0.05; compared to NYX‐2925 + glutamate, *p* > 0.05; Fig. 3a). *Post hoc* analysis also revealed that colocalization of GluN2B‐containing NMDARs with PSD‐95 was unaffected by treatment with other combinations of these ligands (compared to vehicle, *p* > 0.05; Fig. 3a). These data suggest that picomolar NYX‐2925 recruits synaptic GluN2B‐containing NMDARs via a metabotropic‐like mechanism of the NMDAR. Representative immunolabeling is indicated (Fig. 3b).

In order to verify that 1 picomolar NYX‐2925 did not affect calcium transients following activation of NMDARs, calcium imaging was performed using rat primary hippocampal neurons. A low concentration of NMDA (4 μM) was washed onto the cells followed by washout to establish a baseline response for each cell. NYX‐2925 in the presence of 4 μM NMDA was then washed onto the cells to determine if NYX‐2925 enhanced calcium transients beyond NMDA alone. Neither the 1 picomolar nor 1 nanomolar concentration of NYX‐2925 enhanced calcium flux, whereas 30 nanomolar NYX‐2925 resulted in a 15.2% enhancement [*t*(5) = 4.608, *p* = 0.011; Fig. 3c]. A similar dose‐related effect of NYX‐2925 in mediating calcium transients was also observed in primary cortical neurons (Figure S1; 11.5% enhancement at 30 nM, but not 1 pM).

### NYX‐2925 activates trafficking pathways *in vitro*

Next, we sought to determine whether the rapid, differential effects on GluN2 trafficking observed in primary hippocampal cultures following NYX‐2925 treatment were associated with alterations in global proteome dynamics. Thus, we coupled comprehensive proteomics with ontological bioinformatics analysis. A total of 4,860 unique rat proteins (as annotated by ~33 000 unique peptides) were detected across all samples at a false discovery rate of 0.01. When comparing cells treated with 1 pM NYX‐2925 for 30 min with vehicle‐treated cells, 196 significantly differentially expressed (*p* < 0.05) proteins were identified: 122 proteins were up‐regulated and 74 were down‐regulated. When comparing cells treated with 30 nM NYX‐2925 for 30 min with vehicle‐treated cells, 338 significantly differentially expressed (*p* < 0.05) proteins were identified: 261 proteins were up‐regulated and 77 were down‐regulated.

Subsequent ontological analysis revealed that the dose‐dependent proteomic profiles were remarkably distinct in terms of the pathways directly relevant to both receptor trafficking and synaptic plasticity. Tables 1 and 2 summarize these findings, and expanded proteomic results are located in Tables S1 and S2. Exposure of cultures to a metabotropic‐like dose of NYX‐2925 (1 pM) resulted in significant modulation of pathways associated with NMDAR trafficking, and included marked inhibition of clathrin‐mediated endocytosis, (*p* < 3.5 × 10^−4^), EIF2 (9.33 × 10^−10^), and mTOR (2.24 × 10^−8^) signaling as well as an increase in the protein kinase A pathway (*p* < 1.86 × 10^−4^); such modulation could result in increased number and stabilization of membrane receptors.

**Table 1:**
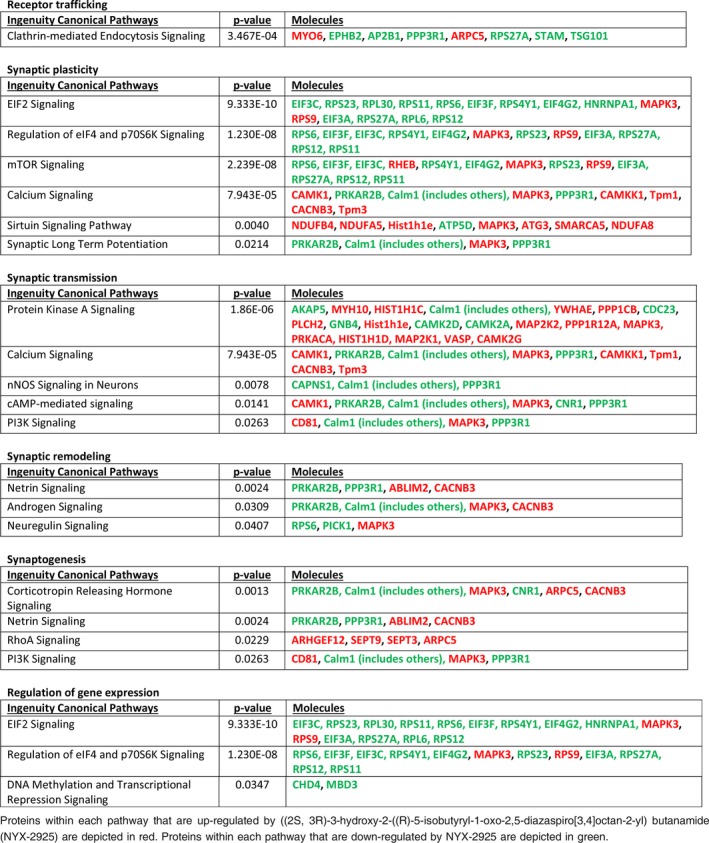
Ontological analysis of differentially expressed hippocampal proteins following treatment with NYX‐2925 (1 pM/30 min)

**Table 2:**
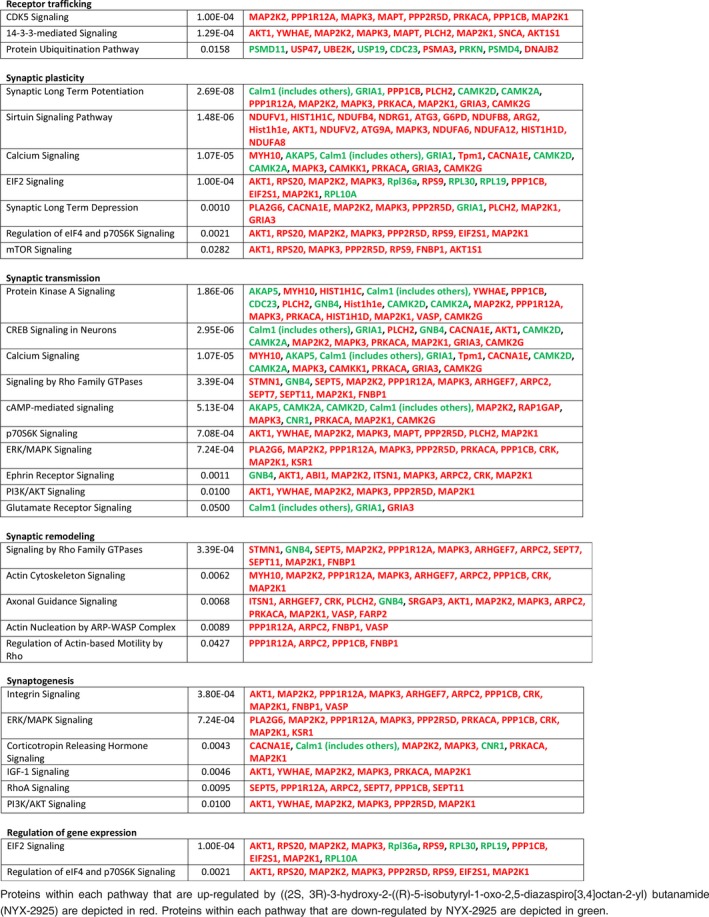
Ontological analysis of differentially expressed hippocampal proteins following treatment with NYX‐2925 (30 nM/30 min)

Conversely, exposure to an ionotropic dose of NYX‐2925 (30 nM) significantly up‐regulated proteins comprising pathways associated with synaptic LTP (*p* < 2.69 × 10^−8^) as well as pathways associated with receptor expression and trafficking, including EIF2 (*p* < 1.00 × 10^−4^), CDK5 (*p* < 1.00 × 10^−4^), 14‐3‐3 (*p* < 1.28 × 10^−4^), and protein kinase A (*p* < 1.86 × 10^−6^); these signaling pathways are associated with synaptic targeting, clustering, and recycling of receptors into and out of the synapse (Table 2).

### NYX‐2925 facilitates the chemLTP‐mediated increase in synaptic GluA1

**Figure 4:**
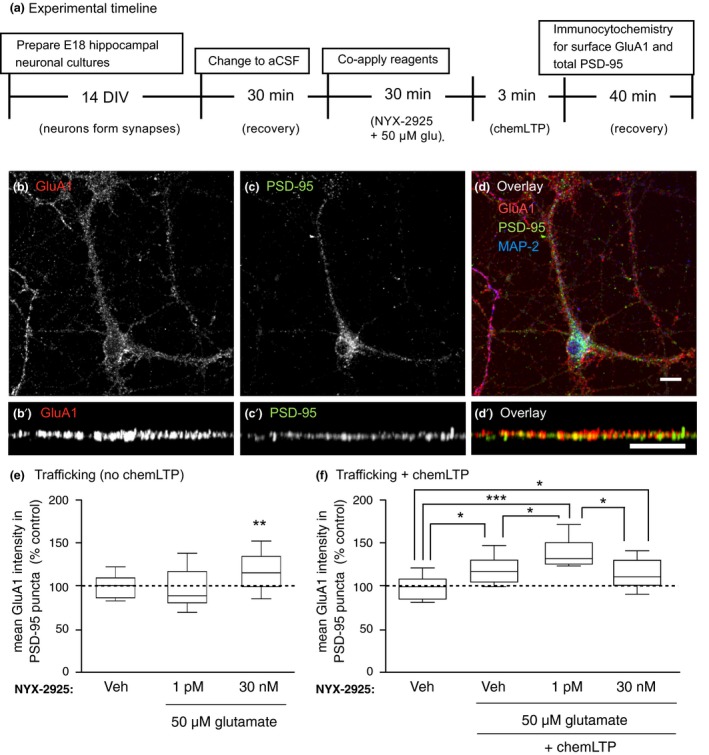
((2S, 3R)‐3‐hydroxy‐2‐((R)‐5‐isobutyryl‐1‐oxo‐2,5‐diazaspiro[3,4]octan‐2‐yl) butanamide (NYX‐2925) pre‐treatment facilitates chemical long‐term potentiation (chemLTP)‐mediated synaptic amino‐3‐hydroxy‐5‐methyl‐4‐isoxazolepropionic acid receptor subunit 1 (GluA1) recruitment. (a) As before, neurons (14 days *in vitro*) were changed to artificial cerebrospinal fluid (aCSF) and allowed to recover for 30 min at 37°C. Next, cells were co‐treated with 1 picomolar NYX‐2925 and 50 μM glutamate and colocalization of GluA1‐containing α‐amino‐3‐hydroxy‐5‐methyl‐4‐isoxazolepropionic acid receptor with post‐synaptic density protein 95 (PSD‐95) quantified. Cells from the same cultures underwent chemLTP stimulation following NYX‐2925/glutamate pre‐treatment. (b–d) Representative cellular immunolabeling of GluA1 and PSD‐95. (b′–d′) Representative dendrites immunolabeled for GluA1 and PSD‐95. (e) Co‐incubation with 1 pM NYX‐2925 and glutamate in the absence of chemLTP did not affect colocalization of GluA1 with PSD‐95. (f) chemLTP stimulation increased colocalization, and this effect was facilitated by 1 pM NYX‐2925/glutamate pre‐treatment. Veh: vehicle. Scale bar = 10 μm. Data represent mean ± SEM ± data spread; the line within each box represents the average, the box itself represents the SEM, and the whiskers indicate the spread of the data. ***p < 0.001, **p < 0.01, *p < 0.05 Tukey *post hoc* analysis, *n* = 14 cells per treatment from two independent cell culture preparations (eight coverslips). MAP2, Microtubule associated protein 2.

Next, we sought to understand the functional impact NYX‐2925 has on neuronal communication. Because frequency‐dependent forms of inducing LTP affect a localized synaptic area, we instead studied a chemical form of LTP (chemLTP), which can stimulate the entire culture (Ahmad *et al.* 2012). The primary readouts of the chemLTP protocol that we used (high glycine, no magnesium) are increased insertion and dendritic clustering of GluA1‐containing AMPARs in addition to more frequent miniature excitatory postsynaptic potentials (Lu *et al.* 2001). Here, synaptic levels of GluA1‐containing AMPARs were studied because they are responsible for the early phase of LTP (Shi *et al.* 2001; Romberg *et al.* 2009), and LTP is absent in GluA1‐null mice (Zamanillo *et al.* 1999). As before, primary hippocampal neurons were treated with vehicle, 1 picomolar, or 30 nanomolar NYX‐2925 in the presence of 50 μM glutamate (Fig. 4a). After removing drug, a subset of cells from each treatment condition continued through the chemLTP protocol (Fig. 4a). Representative immunolabeling of GluA1 and PSD‐95 is shown (Fig. 4b–d).

A one‐way ANOVA followed by *post hoc* analysis found a main effect of NYX‐2925 treatment on GluA1 colocalization with PSD‐95 in the absence of chemLTP [trafficking‐only conditions; *F*(2, 39) = 4.64, *p* = 0.018, *n* = 14 cells per group Fig. 4e]. Furthermore, *post hoc* analysis found a significant effect at 30 nanomolar (*p* = 0.0345), but not 1 picomolar (*p* = 0.5104) NYX‐2925. Following chemLTP, a one‐way anova showed a main effect of chemLTP on GluA1 colocalization with PSD‐95 [*F*(3, 52) = 7.09, *p* = 0.0004, *n* = 14 cells per group). *Post hoc* analysis revealed that this colocalization was increased similarly by pretreatment with either vehicle (compared to vehicle without chemLTP, *p* = 0.0259) or 30 nanomolar NYX‐2925 (compared to vehicle without chemLTP, *p* = 0.0313; Fig. 4f). In contrast, even greater GluA1 colocalization with PSD‐95 was observed after chemLTP when cells were pretreated with 1 picomolar NYX‐2925 (increased colocalization above that caused by either chemLTP alone, *p* = 0.0243, or 30 nanomolar NYX‐2925 followed by chemLTP, *p* = 0.0201; Fig. 4f). Thus, NYX‐2925 concentrations that promote GluN2B colocalization with PSD‐95 were found to also facilitate GluA1 colocalization with PSD‐95 after chemLTP over levels seen with chemLTP alone. In summary, increased NMDAR stimulation, such as that mediated by either high, ionotropic NYX‐2925 concentrations or chemLTP conditions increase synaptic GluA1. In contrast, low NYX‐2925 concentrations that increase synaptic GluN2B do not alter synaptic GluA1 levels on their own, but pre‐treatment with this same low, metabotropic‐like NYX‐2925 concentration facilitates the chemLTP‐mediated recruitment of GluA1 into the synapse. Thus, the NYX‐2925‐mediated recruitment of synaptic GluN2B appears to augment synaptic GluA1 insertion following LTP stimulation. These data agree with prior reports showing that changes in the number or function of NMDARs modulate the LTP induction threshold (Pérez‐Otaño and Ehlers 2005; Abraham 2008).

## Discussion

Here, we report that synaptic levels of GluN2B are increased by picomolar concentrations of the recently developed NMDAR modulator NYX‐2925 *in vitro*, and this effect was replicated *in vivo*. Thus, NYX‐2925 could profoundly impact neuronal communication by facilitating the integration of multiple synaptic inputs via increased GluN2B‐mediated calcium transients and/or receptor number (Scimemi *et al.* 2004). NMDAR activation is required for the NYX‐2925‐mediated colocalization of GluN2B with PSD‐95, as this effect was blocked by the glutamate site antagonist APV. Intriguingly, picomolar NYX‐2925 appears to modulate synaptic GluN2B levels independent of NMDAR ion flux, as the resulting colocalization of GluN2B with PSD‐95 was unaffected by either a NMDAR channel blocker or glycine site antagonist. This antagonist strategy has been used by others to demonstrate ion flux‐independent functions of NMDARs (Nabavi *et al.* 2013; Dore *et al.* 2015). Moreover, picomolar NYX‐2925 had no effect on NMDA‐induced calcium transients in primary neurons. In contrast, nanomolar NYX‐2925 concentrations increased calcium transients, but did not affect colocalization of GluN2B with PSD‐95 unless continuously present in the bath for an extended period (60 min). These concentration‐dependent actions of NYX‐2925 were echoed in the divergent signaling pathways activated at either ionotropic or metabotropic‐like drug concentrations. Lastly, the functional consequence of NYX‐2925 treatment was analyzed via quantification of NMDAR‐mediated insertion of the AMPAR subunit GluA1 into the synapse, which was induced with chemLTP. Because picomolar NYX‐2925 was found to facilitate chemLTP‐induced synaptic GluA1 levels without altering basal GluA1, NYX‐2925 may engage this form of homeostatic plasticity via initially recruiting synaptic GluN2B‐containing NMDARs through a metabotropic‐like mechanism of the NMDAR.

The finding that the NYX‐2925‐mediated increase in synaptic GluN2B was blocked by APV clearly demonstrates that NYX‐2925 operates through NMDARs to exert this effect. Moreover, the observation that neither the channel blocker MK‐801 nor the glycine site antagonist 7CK blocked the NYX‐2925‐mediated increase in synaptic GluN2B supports the idea that NYX‐2925 can act through a metabotropic‐like (ion flux‐independent) mechanism. The inability of MK‐801 and 7CK to prevent long‐term depression (LTD) was previously used to support the conclusion that NMDAR‐mediated LTD (Nabavi *et al.* 2013) and signal transduction across the membrane (Dore *et al.* 2015) can also occur independent of NMDAR ion flux. An additional study found that these antagonists converted the response to a normally ion‐flux‐dependent, LTP‐inducing stimulus into an LTD‐like response (Stein *et al.* 2015). Together with this literature, our findings support the conclusion that NYX‐2925 may modulate synaptic NMDAR levels via metabotropic‐like signaling of the NMDAR. As reviewed by Dore and colleagues (Dore *et al.* 2017), the existence and biological relevance of metabotropic NMDAR signaling is an active area of research with several investigators finding both supportive (Yang *et al.* 2004; Kessels *et al.* 2013; Nabavi *et al.* 2013; Tamburri *et al.* 2013; Dore *et al.* 2015; Kim *et al.* 2015; Stein *et al.* 2015) and contradictory (Babiec *et al.* 2014; Volianskis *et al.* 2015; Sanderson *et al.* 2016) evidence. Among the former, several studies have revealed ion flux‐independent trafficking of GluN2‐containing NMDARs both by NMDAR agonists (Vissel *et al.* 2001; Barria and Malinow 2002) and by co‐agonists (Nong *et al.* 2003; Ferreira *et al.* 2017). Together, these data add to the rich repertoire by which neuronal communication can be shaped by a growing number of NMDAR modulatory mechanisms.

The findings with low picomolar doses of NYX‐2925 reported here add to the growing appreciation that the NMDAR adopts biologically relevant conformational changes independent of channel opening. Other biologically active and therapeutically valuable molecules also function in the low picomolar range. For example, the hepatitis C antiviral pibrentasvir (Mavyret^®^, Abbvie, Chicago, IL, USA) exhibits a low picomolar concentration at which 50% of the population is activated, and was FDA‐approved in 2017 for patients with mild cirrhosis and moderate‐to‐severe kidney disease (Ng *et al.* 2017). Moreover, both neuropeptide S (Raiteri *et al.* 2009) and melatonin (Dubocovich 1983) exert their effects at low picomolar concentrations to modulate neurotransmitter levels.

It is intriguing that pre‐incubation of primary hippocampal neurons with picomolar concentrations of NYX‐2925 increased the capacity of the neuron to recruit GluA1‐containing AMPARs to the synapse following chemLTP. Because picomolar NYX‐2925 did not alter colocalization of GluA1 with PSD‐95 before chemLTP, such low NYX‐2925 concentrations may alter the set‐point for LTP induction by increasing synaptic GluN2B levels. Thus, the rapid acting (within 30 min) and long‐lasting effects of NYX‐2925 (as evident in co‐immunoprecipitates taken 24 h after treatment *in vivo*) may have relevance to homeostatic plasticity processes that are involved in the maintenance and normalization of synaptic function; namely, synaptic scaling and metaplasticity (Abraham and Bear 1996; Pérez‐Otaño and Ehlers 2005). Homeostatic plasticity regulates the magnitude or direction of activity‐dependent plasticity to maintain synaptic scaling within a working range that is typically away from saturation and toxicity. Analogous to the *in vivo* effects observed following administration of NYX‐2925, the enhancement of synaptic scaling mediated by low picomolar concentrations *in vitro* occurs primarily through mobilization of both NMDARs and AMPARs into the synapse. Thus, NYX‐2925 is a novel tool to control the amount of GluN2B, and upon stimulation, GluA1 in the synapse.

**Figure 5:**
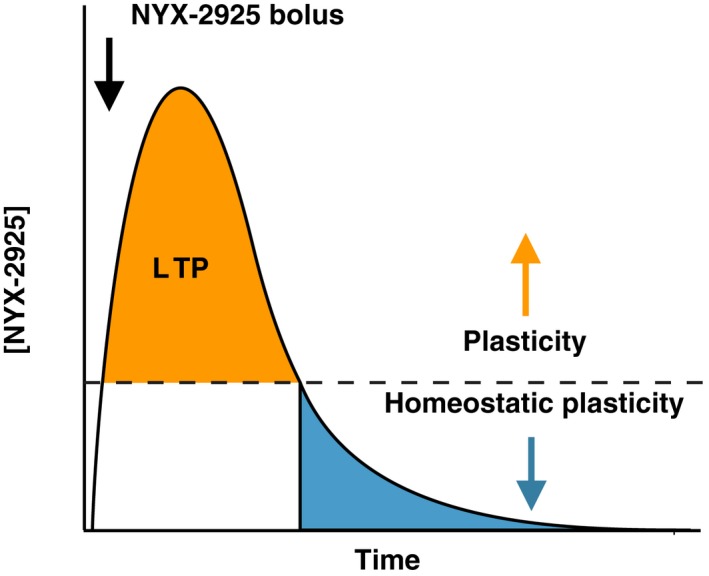
Pharmacokinetic model of ((2S, 3R)‐3‐hydroxy‐2‐((R)‐5‐isobutyryl‐1‐oxo‐2,5‐diazaspiro[3,4]octan‐2‐yl) butanamide (NYX‐2925)‐mediated metaplasticity. Cartoon depicting absorption, metabolism, and elimination of NYX‐2925. Following an oral drug bolus, central levels of NYX‐2925 raise into the concentration range (~44 nanomolar within ~1 h) that facilitates LTP *in vivo* (Khan et al. 2018). With time (> 8 h), drug concentrations fall into the range where homeostatic plasticity is observed (low picomolar), which may account for both the rapid and long‐lasting effects of drug seen *in vivo *following a single treatment. Thus, at some point, brain NYX‐2925 concentrations fall within both the ionotropic and putative metabotropic ranges after oral dosing.

Taken together, our data suggest that NYX‐2925 exhibits two dose‐dependent mechanisms of action (Fig. 5). After drug administration, mid‐nanomolar NYX‐2925 concentrations likely mediate traditional NMDAR functions including ion flux and LTP (Khan *et al.* 2018). NMDARs play critical roles in enduring forms of synaptic plasticity, such as LTP and LTD, and these processes are thought to be a molecular correlate of higher cognitive functions (Bliss and Collingridge 1993). We previously found that NYX‐2925 facilitates LTP at Schaffer collateral‐CA1 synapses when applied to hippocampal slices at 500 nM or following oral administration of a dose (1 mg/kg) that yields 44 nM in the cerebral spinal fluid (Khan *et al.* 2018). This concentration, as noted above, is near the concentration where we observed facilitated calcium transients (Fig. 3), and chemLTP‐like effects on synaptic localization of GluA1 (Fig. 4). This same dose also facilitated novel object recognition and positive emotional learning (Khan *et al.* 2018) as well as reduced neuropathic pain in rats (Ghoreishi‐Haack *et al.* 2018). Thus, mid‐nanomolar NYX‐2925 concentrations facilitate cognition and LTP. As concentrations fall into the low‐picomolar range (> 8 h post‐oral dosing), NYX‐2925 may have a second, ion flux‐independent mechanism of action that engages homeostatic plasticity by lowering the threshold of LTP induction by future stimulation (Fig. 4). This NYX‐2925‐mediated homeostatic plasticity is the conceptual counterpoint of a recent report showing that suppressed AMPAR exocytosis contributes to LTD (Fujii *et al.* 2018). The ability of NYX‐2925 to mediate homeostatic plasticity is further supported by our prior work showing that NYX‐2925 induced metaplasticity *ex vivo* in rats 24 h after oral dosing (Khan *et al.* 2018), a time at which drug concentrations have passed through the range at which metabotropic‐like signaling occurs *in vitro* (Fig. 3). Altogether, these data suggest that NYX‐2925 may exhibit therapeutic utility by facilitating future plasticity (Fig. 4 and Khan *et al.* 2018) via increasing synaptic GluN2B as shown both *in vitro* (Fig. 2) and *in vivo* (Khan *et al.* 2018; Ghoreishi‐Haack *et al.* 2018; Supporting Information). This pharmacokinetic‐based plasticity model (Fig. 5) should be further validated in future work by determining the effect of specific pharmacokinetic phases (e.g. accumulation, distribution, and elimination) on synaptic GluN2B levels and subsequent chemLTP induction thresholds. Nonetheless, the dose‐dependent model of NYX‐2925 action is strongly supported by earlier work showing that lateral diffusion of GluN2B into the synapse engages homeostatic plasticity in intact brain preparations (Zhao *et al.* 2008).

Although the population of neuronal GluN2A and GluN2B was initially thought to be enriched in the synaptic or extrasynaptic membrane, respectively (Kew *et al.* 1998; Tovar and Westbrook 1999; Liu *et al.* 2004), substantial amounts of extrasynaptic GluN2A and synaptic GluN2B have been identified (Luo *et al.* 1997; Groc *et al.* 2006; Thomas *et al.* 2006; Harris and Pettit 2007; Petralia *et al.* 2010), and the relative mobility of these receptor populations are subtype specific, with GluN2B being more mobile than GluN2A (Groc *et al.* 2006; Ferreira *et al.* 2017), and GluN2A less rapidly endocytosed than GluN2B (Lavezzari *et al.* 2004). Thus, synaptic receptor levels are in a state of dynamic equilibrium being routed into and out of the synapse. While it is currently unknown whether NYX‐2925 impacts NMDAR insertion, internalization, or lateralization (Roche *et al.* 2001; Lavezzari *et al.* 2003; Lavezzari *et al.* 2004; Groc *et al.* 2006), our proteomic studies, conducted at a time point when the effect on trafficking is maximal, revealed that metabotropic‐like NYX‐2925 concentrations appear to selectively inhibit clathrin‐mediated endocytosis while facilitating signaling through protein kinase A. Thus, NYX‐2925 appears to increase synaptic GluN2B levels by impairing endocytosis and/or increasing synaptic NMDAR targeting (Crump *et al.* 2001). A recent review elegantly highlighted several signaling mechanisms engaged by ion flux‐independent activity of the NMDAR (de Oca and Balderas 2018); a number of these were identified in our proteomic analysis following treatment with 1 picomolar NYX‐2925.

In stark contrast to the broad inhibition of signaling mediated by picomolar NYX‐2925 (Table 1), nanomolar NYX‐2925 enhanced a number of unique signaling pathways, the most significant of which was LTP (Table 2). These data also reinforce that NMDAR‐mediated calcium influx leads to a multiplicity of effects on receptor‐mediated signaling that can modulate receptor trafficking in complex and unpredictable ways. For example, it is well known that the NMDAR is stabilized in the post‐synaptic membrane via interaction with several scaffolding proteins collectively known as membrane associated guanylate kinases (MAGUKs) (Wenthold *et al.* 2003; Elias and Nicoll 2007). Specifically, the MAGUKs PSD‐95 and SAP102 differentially regulate NMDAR trafficking and localization in a GluN2 subtype‐specific manner (van Zundert *et al.* 2004). While MAGUKs play important roles in anchoring NMDARs in the post‐synaptic density, deletion of these domains does not ablate functional receptor in the synapse. Additional cellular components that also likely modulate receptor trafficking, synaptic retention, and synaptic remodeling were also represented in this dataset. For example, NMDAR scaffolding can also occur via EphrinB, which can increase synaptic retention of GluN2A‐ and GluN2B‐containing NMDARs by extracellular domain interactions (Dalva *et al.* 2000). Additionally, the distinct C‐terminal tails of GluN2A and GluN2B subtypes differentially couple to signaling pathways that can regulate receptor trafficking (Lau and Zukin 2007), dendritic spine remodeling, and synaptic plasticity (Barria and Malinow 2005; Lisman *et al.* 2012). For example, phosphorylation of NMDAR by protein kinase C increases both surface NMDAR levels (Lan *et al.* 2001) and dispersal throughout the dendritic arbor (Fong *et al.* 2002; Groc *et al.* 2004).

NMDAR phosphorylation by the sarcoma (Src) family of protein tyrosine kinases, including Fyn kinase, are also known to modulate NMDAR trafficking both by impairing clathrin‐dependent endocytosis and via enhancing forward GluN2B trafficking (Lavezzari *et al.* 2003; Trepanier *et al.* 2012). Interestingly, Src also appears to regulate PSD‐95 multimerization and subsequent clustering of NMDARs within the synapse via cyclin‐dependent kinase (CDK5) (Morabito et al. 2004). More specifically, inhibition of CDK5 has been shown to increase Src binding to PSD‐95 and subsequent GluN2B phosphorylation of Y1472, which ultimately decreases GluN2B endocytosis (Zhang *et al.* 2008). Thus, our observation that mid‐nanomolar NYX‐2925 concentrations do not increase synaptic GluN2B could be because of CDK5 pathway activation, which under these conditions is hypothesized to promote endocytosis. This mechanism may also underlie the decrease in synaptic GluN2B observed here after prolonged exposure (60 min) to 30 nanomolar NYX‐2925 as well as the decreased immunocontent in whole cell lysates.

Src‐regulated NMDAR trafficking is also modulated by the small GTPase H‐Ras (Thorton *et al.* 2003). NMDAR trafficking via molecular motors (Yunfei *et al.* 2015) and/or small GTPases such as Rho, via Citron, (Furuyashiki *et al.* 1999; Zhang *et al.* 1999) utilize the actin cytoskeleton. Moreover, proteins comprising the phosphoserine/threonine adaptor protein 14‐3‐3 pathway have been demonstrated to promote forward trafficking of GluN2‐containing NMDARs (Chung *et al.* 2015). 14‐3‐3 has been shown to bind the actin cytoskeleton in complex with myosin and calmodulin (Münnich *et al.* 2014). Thus, reorganization of the actin cytoskeleton plays important roles in receptor trafficking during bidirectional synaptic plasticity, including LTP (Okamoto *et al.* 2004; Petralia *et al.* 2009; Kneussel and Wagner 2013). Taken together, our proteomic data underscore that NYX‐2925 may modulate synaptic GluN2B levels by engaging several unique biochemical pathways.

Lastly, although the magnitude of changes in synaptic GluN2B reported here might be considered modest, both the magnitude and time course of changes in synaptic NMDAR are consistent with the body of literature. Using electrophysiological and imaging approaches, the maximal NMDAR exchange between the synaptic and extrasynaptic membrane is about 30% (Tovar and Westbrook 1999; Groc *et al.* 2004; Groc *et al.* 2006). Moreover, these and other studies have shown that NMDARs diffuse laterally across the neuronal surface between synaptic and extrasynaptic membranes within a few minutes (Tovar and Westbrook 1999; Groc *et al.* 2004; Groc and Choquet 2006; Groc *et al.* 2006). The trafficking of distinct GluN2 subtypes into and out of the synaptic membrane is a form of synaptic plasticity that has been associated with a variety of neuropsychiatric and neurological disorders (Pérez‐Otaño and Ehlers 2005; Gardoni *et al.* 2006; Lau and Zukin 2007).

In summary, the data presented here indicate that NYX‐2925 is a novel pharmacological reagent that engages homeostatic plasticity. We found that NYX‐2925 exhibits a dose‐dependent mechanism of action; high concentrations likely activate ionotropic mechanisms whereas low concentrations appear to signal through the NMDAR in a metabotropic‐like manner that increases synaptic levels of ionotropic glutamate receptors. Thus, NYX‐2925 is a powerful tool to interrogate both canonical and unconventional modes of signaling through the NMDAR.

## Author contributions

MAK designed and synthesized NYX‐2925. MSB, LPC, RAK, and JRM designed experiments. MSB, LPC, MES, JAS, and SUS ran experiments. MSB, LPC, KL, SUS, RAK, and JRM analyzed and interpreted experiments. MSB, LPC, KL, RAK, and JRM wrote the paper.

## Supporting information


**Figure S1.** Effects of ((2S, 3R)‐3‐hydroxy‐2‐((R)‐5‐isobutyryl‐1‐oxo‐2,5‐diazaspiro[3,4]octan‐2‐yl) butanamide on calcium transients following *N*‐methyl‐d‐aspartate receptor activation.
**Table S1.** Complete ontological analysis of differentially expressed hippocampal proteins following treatment with ((2S, 3R)‐3‐hydroxy‐2‐((R)‐5‐isobutyryl‐1‐oxo‐2,5‐diazaspiro[3,4]octan‐2‐yl) butanamide (1 pM/30 min).
**Table S2.** Complete ontological analysis of differentially expressed hippocampal proteins following treatment with ((2S, 3R)‐3‐hydroxy‐2‐((R)‐5‐isobutyryl‐1‐oxo‐2,5‐diazaspiro[3,4]octan‐2‐yl) butanamide (30 nM/30 min).
**Data S1.** Supplementary result.
