# Supplementary material for: NYX‐2925 induces metabotropic N‐methyl‐d‐aspartate receptor (NMDAR) signaling that enhances synaptic NMDAR and α‐amino‐3‐hydroxy‐5‐methyl‐4‐isoxazolepropionic acid receptor
Source: J Neurochem. 2019 Oct 16;152(5):523–41. doi: 10.1111/jnc.14845 (PMC7065110; doi:10.1111/jnc.14845)

Title: NYX-2925 induces metabotropic NMDAR signaling that enhances synaptic NMDAR and AMPAR (**84 Characters**)

M. Scott Bowers[1,2], Luisa P. Cacheaux[2], Srishti U. Sahu[1], Mary E. Schmidt[2], Joseph A. Sennello[2], Katherine Leaderbrand[2], Roger A. Kroes[1,2], Joseph R. Moskal[1,2*]

[1.] Falk Center for Molecular Therapeutics, Biomedical Engineering, Northwestern University, Evanston, IL 60201, USA;
[2.] Aptinyx, Inc., Evanston, IL 60201, USA

Supplementary Material

1. Supplementary Results

<u>1.1 NYX-2925 mediates cortical neuron calcium transients in vitro.</u>

Calcium imaging was also performed in rat primary cortical neurons. Identical to our experiment in primary hippocampal neurons, a low concentration of NMDA (4 µM) was washed onto the cells followed by washout to establish a baseline for each cell. NYX-2925 in the presence of 4 µM NMDA was then washed onto the cells to determine if NYX-2925 enhanced calcium transients beyond NMDA alone. The 1 picomolar concentration of NYX-2925 did not enhance calcium flux whereas 30 nanomolar NYX-2925 resulted in an 11.5% enhancement ($t(6)=2.40$, $p = 0.05$; **Figure S1**). These data mirror effects observed in primary hippocampal neurons (**Figure 3C**).

<u>1.2 NYX-2925 activates trafficking pathways in vivo.</u>

To determine if NYX-2925 affects synaptic NMDARs in vivo, rats were treated with NYX-2925 (1 mg/kg, P.O.) and prefrontal cortex collected 24 h later for co-immunoprecipitation and proteomic analysis. Both GluN2A and GluN2B increased in PSD-95 co-immunoprecipitates by 2.72 ($p = 0.02$) and 2.19 fold ($p = 0.009$), respectively.

2. Supplementary Legends

Figure S1. Effects of NYX-2925 on calcium transients following NMDAR activation. Primary cortical neurons were loaded with Fluo-4-AM, and calcium imaging performed. After a baseline measurement, 4 μM NMDA was washed onto the cells for 1 min followed by a 2 min washout. NYX-2925 + NMDA was then washed onto the cells to determine if NYX-2925 enhanced NMDA receptor mediated calcium transients. Two independent culture preparations were used. Data represent mean ± SEM; Student's t-Test $t(6)=2.40$, $p = 0.05$, $n = 6$ cover slips.

Supplementary Table 1. Complete ontological analysis of differentially expressed hippocampal proteins following treatment with NYX-2925 (1 pM/3 0min)

Ingenuity Canonical Pathways	p-value	Molecules
EIF2 Signaling	9.333E-10	EIF3C, RPS23, RPL30, RPS11, RPS6, EIF3F, RPS4Y1, EIF4G2, HNRNPA1, MAPK3, RPS9, EIF3A, RPS27A, RPL6, RPS12
Regulation of eIF4 and p70S6K Signaling	1.230E-08	RPS6, EIF3F, EIF3C, RPS4Y1, EIF4G2, MAPK3, RPS23, RPS9, EIF3A, RPS27A, RPS12, RPS11
mTOR Signaling	2.239E-08	RPS6, EIF3F, EIF3C, RHEB, RPS4Y1, EIF4G2, MAPK3, RPS23, RPS9, EIF3A, RPS27A, RPS12, RPS11
Calcium Signaling	7.943E-05	CAMK1, PRKAR2B, Calm1 (includes others), MAPK3, PPP3R1, CAMKK1, Tpm1, CACNB3, Tpm3
Clathrin-mediated Endocytosis Signaling	3.467E-04	MYO6, EPHB2, AP2B1, PPP3R1, ARPC5, RPS27A, STAM, TSG101
Opioid Signaling Pathway	0.0013	MAP2K6, CAMK1, PRKAR2B, Calm1 (includes others), MAPK3, AP2B1, PPP3R1, CACNB3
Corticotropin Releasing Hormone Signaling	0.0013	PRKAR2B, Calm1 (includes others), MAPK3, CNR1, ARPC5, CACNB3
Huntington's Disease Signaling	0.0015	CAPNS1, ATP5D, YKT6, MAPK3, HSPA1A/HSPA1B, RPS27A, TCERG1, NAPB
Netrin Signaling	0.0024	PRKAR2B, PPP3R1, ABLIM2, CACNB3
Role of NFAT in Cardiac Hypertrophy	0.0030	MAP2K6, CAMK1, PRKAR2B, Calm1 (includes others), MAPK3, PPP3R1, CACNB3
Melatonin Signaling	0.0035	MAP2K6, PRKAR2B, Calm1 (includes others), MAPK3
Sirtuin Signaling Pathway	0.0040	NDUFB4, NDUFA5, Hist1h1e, ATP5D, MAPK3, ATG3, SMARCA5, NDUFA8
nNOS Signaling in Skeletal Muscle Cells	0.0054	Calm1 (includes others), CACNB3, DAG1
nNOS Signaling in Neurons	0.0078	CAPNS1, Calm1 (includes others), PPP3R1
Breast Cancer Regulation by Stathmin1	0.0089	ARHGEF12, CAMK1, PRKAR2B, Calm1 (includes others), MAPK3, TSG101
Phagosome Maturation	0.0093	VPS28, YKT6, TSG101, NAPB, PRDX6
Amyloid Processing	0.0098	CAPNS1, PRKAR2B, MAPK3
RANK Signaling in Osteoclasts	0.0117	MAP2K6, Calm1 (includes others), MAPK3, PPP3R1
cAMP-mediated signaling	0.0141	CAMK1, PRKAR2B, Calm1 (includes others), MAPK3, CNR1, PPP3R1
GNRH Signaling	0.0145	MAP2K6, PRKAR2B, Calm1 (includes others), MAPK3, CACNB3
Oxidative Phosphorylation	0.0148	NDUFB4, NDUFA5, ATP5D, NDUFA8
Mitochondrial Dysfunction	0.0166	NDUFB4, NDUFA5, ATP5D, GPX4, NDUFA8
Spermidine Biosynthesis I	0.0174	SRM
Glutathione Redox Reactions I	0.0182	GPX4, PRDX6
Synaptic Long Term Potentiation	0.0214	PRKAR2B, Calm1 (includes others), MAPK3, PPP3R1
fMLP Signaling in Neutrophils	0.0219	Calm1 (includes others), MAPK3, PPP3R1, ARPC5
RhoA Signaling	0.0229	ARHGEF12, SEPT9, SEPT3, ARPC5
Chemokine Signaling	0.0234	CAMK1, Calm1 (includes others), MAPK3
PI3K Signaling	0.0263	CD81, Calm1 (includes others), MAPK3, PPP3R1
Androgen Signaling	0.0309	PRKAR2B, Calm1 (includes others), MAPK3, CACNB3
Regulation of IL-2 Expression in Activated and Anergic T Lymphocytes	0.0324	Calm1 (includes others), MAPK3, PPP3R1
Phenylethylamine Degradation I	0.0339	ALDH3A2
Methylmalonyl Pathway	0.0339	PCCA
DNA Methylation and Transcriptional Repression Signaling	0.0347	CHD4, MBD3
α-Adrenergic Signaling	0.0398	PRKAR2B, Calm1 (includes others), MAPK3
Neuregulin Signaling	0.0407	RPS6, PICK1, MAPK3

Tetrahydrofolate Salvage from 5,10-methenyltetrahydrofolate	0.0427	**MTHFD1**
2-oxobutanoate Degradation I	0.0427	**PCCA**
Folate Polyglutamylation	0.0427	**MTHFD1**
Mechanisms of Viral Exit from Host Cells	0.0490	**VPS28**, **TSG101**
Salvage Pathways of Pyrimidine Ribonucleotides	0.0490	**MAP2K6**, **UPRT**, **MAPK3**
GABA Receptor Signaling	0.0490	**AP2B1, RPS27A, CACNB3**

Supplementary Table 2. Complete ontological analysis of differentially expressed hippocampal proteins following treatment with NYX-2925 (30 nM/30 min)

Ingenuity Canonical Pathways	p-value	Molecules
Synaptic Long Term Potentiation	2.69E-08	Calm1 (includes others), GRIA1, PPP1CB, PLCH2, CAMK2D, CAMK2A, PPP1R12A, MAP2K2, MAPK3, PRKACA, MAP2K1, GRIA3, CAMK2G
Melatonin Signaling	8.91E-08	MAP2K6, CAMK2A, CAMK2D, Calm1 (includes others), MAP2K2, MAPK3, PRKACA, PLCH2, MAP2K1, CAMK2G
Mitochondrial Dysfunction	2.19E-07	NDUFV1, NDUFB4, COX17, NDUFB8, ATP5S, GSR, NDUFV2, NDUFA6, CAT, COX5A, NDUFA12, PRKN, SNCA, NDUFA8
Breast Cancer Regulation by Stathmin1	3.55E-07	Calm1 (includes others), PPP2R5D, ARHGEF7, PPP1CB, TSG101, STMN1, GNB4, CAMK2D, CAMK2A, PPP1R12A, MAP2K2, MAPK3, PRKACA, MAP2K1, CAMK2G
Chemokine Signaling	8.71E-07	CAMK2A, CAMK2D, Calm1 (includes others), MAP2K2, PPP1R12A, MAPK3, PPP1CB, MAP2K1, CAMK2G
Sirtuin Signaling Pathway	1.48E-06	NDUFV1, HIST1H1C, NDUFB4, NDRG1, ATG3, G6PD, NDUFB8, ARG2, Hist1h1e, AKT1, NDUFV2, ATG9A, MAPK3, NDUFA6, NDUFA12, HIST1H1D, NDUFA8
Protein Kinase A Signaling	1.86E-06	AKAP5, MYH10, HIST1H1C, Calm1 (includes others), YWHAE, PPP1CB, CDC23, PLCH2, GNB4, Hist1h1e, CAMK2D, CAMK2A, MAP2K2, PPP1R12A, MAPK3, PRKACA, HIST1H1D, MAP2K1, VASP, CAMK2G
CREB Signaling in Neurons	2.95E-06	Calm1 (includes others), GRIA1, PLCH2, GNB4, CACNA1E, AKT1, CAMK2D, CAMK2A, MAP2K2, MAPK3, PRKACA, MAP2K1, GRIA3, CAMK2G
Role of NFAT in Cardiac Hypertrophy	4.27E-06	MAP2K6, AKAP5, Calm1 (includes others), PLCH2, GNB4, CACNA1E, AKT1, CAMK2D, CAMK2A, MAP2K2, MAPK3, PRKACA, MAP2K1, CAMK2G
Oxidative Phosphorylation	4.37E-06	NDUFV1, NDUFB4, COX17, NDUFV2, NDUFA6, COX5A, NDUFA12, NDUFB8, ATP5S, NDUFA8
Calcium Signaling	1.07E-05	MYH10, AKAP5, Calm1 (includes others), GRIA1, Tpm1, CACNA1E, CAMK2D, CAMK2A, MAPK3, CAMKK1, PRKACA, GRIA3, CAMK2G
NRF2-mediated Oxidative Stress Response	2.75E-05	MAP2K6, GSR, AKT1, MAP2K2, MAPK3, CAT, UBE2K, DNAJA2, DNAJB2, MAP2K1, CLPP, GSTP1
Thrombin Signaling	4.68E-05	GNB4, CAMK2A, CAMK2D, AKT1, MAP2K2, PPP1R12A, MAPK3, PPP1CB, PLCH2, MAP2K1, FNBP1, CAMK2G
GM-CSF Signaling	9.12E-05	CAMK2A, CAMK2D, AKT1, MAP2K2, MAPK3, MAP2K1, CAMK2G
CDK5 Signaling	1.00E-04	MAP2K2, PPP1R12A, MAPK3, MAPT, PPP2R5D, PRKACA, PPP1CB, MAP2K1
EIF2 Signaling	1.00E-04	AKT1, RPS20, MAP2K2, MAPK3, Rpl36a, RPS9, RPL30, RPL19, PPP1CB, EIF2S1, MAP2K1, RPL10A
PI3K Signaling in B Lymphocytes	1.23E-04	CAMK2A, CAMK2D, AKT1, Calm1 (includes others), MAP2K2, MAPK3, PLCH2, MAP2K1, CAMK2G
14-3-3-mediated Signaling	1.29E-04	AKT1, YWHAE, MAP2K2, MAPK3, MAPT, PLCH2, MAP2K1, SNCA, AKT1S1
GNRH Signaling	1.55E-04	MAP2K6, CAMK2A, CAMK2D, CACNA1E, Calm1 (includes others), MAP2K2, MAPK3, PRKACA, MAP2K1, CAMK2G
Insulin Receptor Signaling	2.24E-04	AKT1, MAP2K2, PPP1R12A, MAPK3, PRKACA, PPP1CB, CRK, VAMP2, MAP2K1
Opioid Signaling Pathway	2.34E-04	MAP2K6, CAMK2A, CAMK2D, AKT1, CACNA1E, Calm1 (includes others), MAP2K2, MAPK3, CLTC, PRKACA, MAP2K1, CAMK2G
Nitric Oxide Signaling in the Cardiovascular System	2.51E-04	AKT1, CACNA1E, Calm1 (includes others), MAP2K2, MAPK3, PRKACA, ARG2, MAP2K1
Glioma Signaling	2.63E-04	CAMK2A, CAMK2D, AKT1, Calm1 (includes others), MAP2K2, MAPK3, MAP2K1, CAMK2G
Neuropathic Pain Signaling In Dorsal Horn Neurons	2.82E-04	CAMK2A, CAMK2D, MAPK3, GRIA1, PRKACA, PLCH2, CAMK2G, GRIA3
Signaling by Rho Family GTPases	3.39E-04	STMN1, GNB4, SEPT5, MAP2K2, PPP1R12A, MAPK3, ARHGEF7, ARPC2, SEPT7, SEPT11, MAP2K1, FNBP1
Integrin Signaling	3.80E-04	AKT1, MAP2K2, PPP1R12A, MAPK3, ARHGEF7, ARPC2, PPP1CB, CRK, MAP2K1, FNBP1, VASP
Fcγ Receptor-mediated Phagocytosis in Macrophages and Monocytes	4.17E-04	MYO5A, PLA2G6, AKT1, MAPK3, ARPC2, CRK, VASP
Salvage Pathways of Pyrimidine Ribonucleotides	4.79E-04	MAP2K6, UPRT, MAP2K2, MAPK3, NME2, AK4, MAP2K1
cAMP-mediated signaling	5.13E-04	AKAP5, CAMK2A, CAMK2D, Calm1 (includes others), MAP2K2, RAP1GAP, MAPK3, CNR1, PRKACA, MAP2K1, CAMK2G

Role of MAPK Signaling in the Pathogenesis of Influenza	6.31E-04	**MAP2K6, PLA2G6, AKT1, MAP2K2, MAPK3, MAP2K1**
CCR3 Signaling in Eosinophils	6.46E-04	**PLA2G6, GNB4, Calm1 (includes others), MAP2K2, PPP1R12A, MAPK3, PPP1CB, MAP2K1**
p70S6K Signaling	7.08E-04	**AKT1, YWHAE, MAP2K2, MAPK3, MAPT, PPP2R5D, PLCH2, MAP2K1**
Cardiac Hypertrophy Signaling	7.08E-04	**MAP2K6, GNB4, AKT1, CACNA1E, Calm1 (includes others), MAP2K2, MAPK3, PRKACA, PLCH2, MAP2K1, FNBP1**
ERK/MAPK Signaling	7.24E-04	**PLA2G6, MAP2K2, PPP1R12A, MAPK3, PPP2R5D, PRKACA, PPP1CB, CRK, MAP2K1, KSR1**
Amyloid Processing	8.51E-04	**AKT1, MAPK3, MAPT, PRKACA, MARK1**
Synaptic Long Term Depression	0.0010	**PLA2G6, CACNA1E, MAP2K2, MAPK3, PPP2R5D, GRIA1, PLCH2, MAP2K1, GRIA3**
Ephrin Receptor Signaling	0.0011	**GNB4, AKT1, ABI1, MAP2K2, ITSN1, MAPK3, ARPC2, CRK, MAP2K1**
Xenobiotic Metabolism Signaling	0.0012	**MAP2K6, CAMK2A, CAMK2D, MAP2K2, MAPK3, PPP2R5D, CAT, HS2ST1, ALDH18A1, MAP2K1, GSTP1, CAMK2G**
Superpathway of Citrulline Metabolism	0.0012	**ALDH18A1, ARG2, ASL**
Parkinson's Signaling	0.0015	**SEPT5, PRKN, SNCA**
Leptin Signaling in Obesity	0.0016	**AKT1, MAP2K2, MAPK3, PRKACA, PLCH2, MAP2K1**
α-Adrenergic Signaling	0.0017	**GNB4, Calm1 (includes others), MAP2K2, MAPK3, PRKACA, MAP2K1**
B Cell Receptor Signaling	0.0020	**MAP2K6, CAMK2A, CAMK2D, AKT1, Calm1 (includes others), MAP2K2, MAPK3, MAP2K1, CAMK2G**
Molecular Mechanisms of Cancer	0.0020	**MAP2K6, ARHGEF7, CRK, SYNGAP1, RASGRF2, AKT1, CAMK2D, CAMK2A, MAP2K2, MAPK3, PRKACA, MAP2K1, FNBP1, CAMK2G**
Regulation of eIF4 and p70S6K Signaling	0.0021	**AKT1, RPS20, MAP2K2, MAPK3, PPP2R5D, RPS9, EIF2S1, MAP2K1**
Production of Nitric Oxide and Reactive Oxygen Species in Macrophages	0.0022	**AKT1, PPP1R12A, MAPK3, PPP2R5D, CAT, PPP1CB, ARG2, MAP2K1, FNBP1**
ILK Signaling	0.0025	**MAP2K6, MYH10, AKT1, PPP1R12A, MAPK3, PPP2R5D, FERMT2, RSU1, FNBP1**
G-Protein Coupled Receptor Signaling	0.0025	**SYNGAP1, CAMK2A, CAMK2D, AKT1, MAP2K2, RAP1GAP, MAPK3, CNR1, PRKACA, MAP2K1, CAMK2G**
Dopamine-DARPP32 Feedback in cAMP Signaling	0.0028	**CACNA1E, Calm1 (includes others), PPP1R12A, PPP2R5D, CAMKK1, PRKACA, PPP1CB, PLCH2**
Granzyme A Signaling	0.0029	**HIST1H1C, Hist1h1e, HIST1H1D**
Phospholipase C Signaling	0.0029	**PLA2G6, GNB4, Calm1 (includes others), MAP2K2, PPP1R12A, MAPK3, ARHGEF7, PPP1CB, MAP2K1, FNBP1**
Mechanisms of Viral Exit from Host Cells	0.0029	**SNF8, CHMP4B, LMNB1, TSG101**
Urea Cycle	0.0030	**ARG2, ASL**
Cellular Effects of Sildenafil (Viagra)	0.0031	**MYH10, CACNA1E, Calm1 (includes others), PPP1R12A, PRKACA, PPP1CB, PLCH2**
FAK Signaling	0.0032	**AKT1, MAP2K2, MAPK3, ARHGEF7, CRK, MAP2K1**
P2Y Purigenic Receptor Signaling Pathway	0.0035	**GNB4, AKT1, MAP2K2, MAPK3, PRKACA, PLCH2, MAP2K1**
RANK Signaling in Osteoclasts	0.0038	**MAP2K6, AKT1, Calm1 (includes others), MAP2K2, MAPK3, MAP2K1**
VEGF Signaling	0.0040	**AKT1, YWHAE, MAP2K2, MAPK3, EIF2S1, MAP2K1**
Corticotropin Releasing Hormone Signaling	0.0043	**CACNA1E, Calm1 (includes others), MAP2K2, MAPK3, CNR1, PRKACA, MAP2K1**
Cardiac β-adrenergic Signaling	0.0045	**AKAP5, GNB4, CACNA1E, PPP1R12A, PPP2R5D, PRKACA, PPP1CB**
Chronic Myeloid Leukemia Signaling	0.0046	**AKT1, MAP2K2, MAPK3, CTBP2, CRK, MAP2K1**
IGF-1 Signaling	0.0046	**AKT1, YWHAE, MAP2K2, MAPK3, PRKACA, MAP2K1**
Neurotrophin/TRK Signaling	0.0052	**MAP2K6, AKT1, MAP2K2, MAPK3, MAP2K1**
Gαs Signaling	0.0055	**GNB4, MAP2K2, MAPK3, CNR1, PRKACA, MAP2K1**
Cancer Drug Resistance By Drug Efflux	0.0055	**AKT1, MAP2K2, MAPK3, MAP2K1**
Role of PI3K/AKT Signaling in the Pathogenesis of Influenza	0.0059	**AKT1, MAP2K2, MAPK3, CRK, MAP2K1**

Actin Cytoskeleton Signaling	0.0062	MYH10, MAP2K2, PPP1R12A, MAPK3, ARHGEF7, ARPC2, PPP1CB, CRK, MAP2K1
Axonal Guidance Signaling	0.0068	ITSN1, ARHGEF7, CRK, PLCH2, GNB4, SRGAP3, AKT1, MAP2K2, MAPK3, ARPC2, PRKACA, MAP2K1, VASP, FARP2
Citrulline Biosynthesis	0.0071	ALDH18A1, ARG2
Renal Cell Carcinoma Signaling	0.0072	AKT1, MAP2K2, MAPK3, CRK, MAP2K1
Gap Junction Signaling	0.0079	AKT1, MAP2K2, MAPK3, GRIA1, PRKACA, PLCH2, MAP2K1, GRIA3
Fc Epsilon RI Signaling	0.0079	MAP2K6, PLA2G6, AKT1, MAP2K2, MAPK3, MAP2K1
IL-17 Signaling	0.0079	MAP2K6, AKT1, MAP2K2, MAPK3, MAP2K1
Melanoma Signaling	0.0083	AKT1, MAP2K2, MAPK3, MAP2K1
Actin Nucleation by ARP-WASP Complex	0.0089	PPP1R12A, ARPC2, FNBP1, VASP
Neuregulin Signaling	0.0091	AKT1, MAP2K2, MAPK3, CRK, MAP2K1
VEGF Family Ligand-Receptor Interactions	0.0091	PLA2G6, AKT1, MAP2K2, MAPK3, MAP2K1
fMLP Signaling in Neutrophils	0.0093	GNB4, Calm1 (includes others), MAP2K2, MAPK3, ARPC2, MAP2K1
Gαq Signaling	0.0093	GNB4, AKT1, Calm1 (includes others), MAP2K2, MAPK3, MAP2K1, FNBP1
RhoA Signaling	0.0095	SEPT5, PPP1R12A, ARPC2, SEPT7, PPP1CB, SEPT11
PI3K/AKT Signaling	0.0100	AKT1, YWHAE, MAP2K2, MAPK3, PPP2R5D, MAP2K1
FGF Signaling	0.0105	MAP2K6, AKT1, MAPK3, CRK, MAP2K1
CXCR4 Signaling	0.0107	GNB4, AKT1, MAP2K2, MAPK3, CRK, MAP2K1, FNBP1
Pentose Phosphate Pathway	0.0107	TALDO1, G6PD
4-1BB Signaling in T Lymphocytes	0.0110	MAP2K2, MAPK3, MAP2K1
Tight Junction Signaling	0.0112	MYH10, AKT1, PPP2R5D, MARK2, PRKACA, CSTF2, VASP
Ceramide Signaling	0.0115	AKT1, MAPK3, PPP2R5D, MAP2K1, KSR1
Acute Myeloid Leukemia Signaling	0.0115	MAP2K6, AKT1, MAP2K2, MAPK3, MAP2K1
Oncostatin M Signaling	0.0132	MAP2K2, MAPK3, MAP2K1
Sumoylation Pathway	0.0132	SAE1, CTBP2, RANGAP1, SNCA, FNBP1
HMGB1 Signaling	0.0132	MAP2K6, AKT1, MAP2K2, MAPK3, MAP2K1, FNBP1
CNTF Signaling	0.0132	AKT1, MAP2K2, MAPK3, MAP2K1
Germ Cell-Sertoli Cell Junction Signaling	0.0135	MAP2K6, EPN2, AKT1, MAP2K2, MAPK3, MAP2K1, FNBP1
Prostate Cancer Signaling	0.0138	AKT1, MAP2K2, MAPK3, MAP2K1, GSTP1
Endometrial Cancer Signaling	0.0141	AKT1, MAP2K2, MAPK3, MAP2K1
IL-2 Signaling	0.0141	AKT1, MAP2K2, MAPK3, MAP2K1
Melanocyte Development and Pigmentation Signaling	0.0141	MAP2K2, MAPK3, PRKACA, CRK, MAP2K1
ErbB Signaling	0.0141	MAP2K6, AKT1, MAP2K2, MAPK3, MAP2K1
Pyridoxal 5'-phosphate Salvage Pathway	0.0148	MAP2K6, MAP2K2, MAPK3, MAP2K1
Protein Ubiquitination Pathway	0.0158	PSMD11, USP47, UBE2K, USP19, CDC23, PSMA3, PRKN, PSMD4, DNAJB2
Cholecystokinin/Gastrin-mediated Signaling	0.0158	MAP2K6, MAP2K2, MAPK3, MAP2K1, FNBP1
Role of Macrophages, Fibroblasts and Endothelial Cells in Rheumatoid Arthritis	0.0162	MAP2K6, CAMK2A, CAMK2D, AKT1, Calm1 (includes others), MAP2K2, MAPK3, PLCH2, MAP2K1, CAMK2G
Role of IL-17A in Arthritis	0.0182	MAP2K6, MAP2K2, MAPK3, MAP2K1
ErbB2-ErbB3 Signaling	0.0182	AKT1, MAP2K2, MAPK3, MAP2K1
Role of NFAT in Regulation of the Immune Response	0.0195	AKAP5, GNB4, AKT1, Calm1 (includes others), MAP2K2, MAPK3, MAP2K1
Epithelial Adherens Junction Signaling	0.0200	MYH10, EPN2, AKT1, ARPC2, CRK, FARP2

Thyroid Cancer Signaling	0.0204	MAP2K2, MAPK3, MAP2K1
ErbB4 Signaling	0.0209	AKT1, MAP2K2, MAPK3, MAP2K1
Role of PKR in Interferon Induction and Antiviral Response	0.0214	MAP2K6, AKT1, EIF2S1
Ephrin B Signaling	0.0219	GNB4, ABI1, ITSN1, MAPK3
Amyotrophic Lateral Sclerosis Signaling	0.0229	CACNA1E, HECW1, GRIA1, CAT, GRIA3
Telomerase Signaling	0.0229	AKT1, MAP2K2, MAPK3, PPP2R5D, MAP2K1
UVC-Induced MAPK Signaling	0.0245	MAP2K2, MAPK3, MAP2K1
IL-15 Signaling	0.0245	AKT1, MAP2K2, MAPK3, MAP2K1
Antiproliferative Role of Somatostatin Receptor 2	0.0245	GNB4, MAP2K2, MAPK3, MAP2K1
BMP signaling pathway	0.0245	MAP2K2, MAPK3, PRKACA, MAP2K1
IL-8 Signaling	0.0257	GNB4, AKT1, MAP2K2, MAPK3, MAP2K1, FNBP1, VASP
G Beta Gamma Signaling	0.0257	GNB4, AKT1, CACNA1E, MAPK3, PRKACA
Non-Small Cell Lung Cancer Signaling	0.0257	AKT1, MAP2K2, MAPK3, MAP2K1
Dopamine Receptor Signaling	0.0257	PPP1R12A, PPP2R5D, PRKACA, PPP1CB
IL-17A Signaling in Airway Cells	0.0269	AKT1, MAP2K2, MAPK3, MAP2K1
Role of IL-17F in Allergic Inflammatory Airway Diseases	0.0275	MAP2K2, MAPK3, MAP2K1
mTOR Signaling	0.0282	AKT1, RPS20, MAPK3, PPP2R5D, RPS9, FNBP1, AKT1S1
CD40 Signaling	0.0282	MAP2K6, MAP2K2, MAPK3, MAP2K1
Regulation of IL-2 Expression in Activated and Anergic T Lymphocytes	0.0295	Calm1 (includes others), MAP2K2, MAPK3, MAP2K1
Erythropoietin Signaling	0.0302	AKT1, MAP2K2, MAPK3, MAP2K1
Gαi Signaling	0.0309	GNB4, RAP1GAP, MAPK3, CNR1, PRKACA
Glioblastoma Multiforme Signaling	0.0316	AKT1, MAP2K2, MAPK3, PLCH2, MAP2K1, FNBP1
NGF Signaling	0.0316	AKT1, MAP2K2, MAPK3, CRK, MAP2K1
IL-3 Signaling	0.0331	AKT1, MAP2K2, MAPK3, MAP2K1
JAK/Stat Signaling	0.0331	AKT1, MAP2K2, MAPK3, MAP2K1
iCOS-iCOSL Signaling in T Helper Cells	0.0339	CAMK2A, CAMK2D, AKT1, Calm1 (includes others), CAMK2G
FLT3 Signaling in Hematopoietic Progenitor Cells	0.0372	AKT1, MAP2K2, MAPK3, MAP2K1
LPS-stimulated MAPK Signaling	0.0380	MAP2K6, MAP2K2, MAPK3, MAP2K1
HIPPO signaling	0.0380	YWHAE, PPP1R12A, PPP2R5D, PPP1CB
TGF-β Signaling	0.0380	MAP2K6, MAP2K2, MAPK3, MAP2K1
Estrogen Receptor Signaling	0.0389	MAP2K2, THRAP3, MAPK3, CTBP2, MAP2K1
IL-6 Signaling	0.0389	MAP2K6, AKT1, MAP2K2, MAPK3, MAP2K1
CD27 Signaling in Lymphocytes	0.0417	MAP2K6, MAP2K2, MAP2K1
Regulation of Actin-based Motility by Rho	0.0427	PPP1R12A, ARPC2, PPP1CB, FNBP1
Apoptosis Signaling	0.0427	MAP2K2, MAPK3, LMNA, MAP2K1
PDGF Signaling	0.0427	MAP2K2, MAPK3, CRK, MAP2K1
Methylglyoxal Degradation I	0.0427	HAGH
Biotin-carboxyl Carrier Protein Assembly	0.0427	ACACA
Pyrimidine Deoxyribonucleotides De Novo Biosynthesis I	0.0437	NME2, AK4
CD28 Signaling in T Helper Cells	0.0437	AKT1, Calm1 (includes others), MAP2K2, ARPC2, MAP2K1

Sertoli Cell-Sertoli Cell Junction Signaling	0.0457	**EPN2, AKT1, MAP2K2, MAPK3, PRKACA, MAP2K1**
IL-22 Signaling	0.0468	**AKT1, MAPK3**
Glutathione Redox Reactions I	0.0468	**GSR**, **GSTP1**
PPARα/RXRα Activation	0.0479	**MAP2K6, MAP2K2, MAPK3, PRKACA, PLCH2, MAP2K1**
Androgen Signaling	0.0500	**GNB4**, **CACNA1E**, **Calm1 (includes others), MAPK3**, **PRKACA**
Glutamate Receptor Signaling	0.0500	**Calm1 (includes others), GRIA1**, **GRIA3**

FIGURE S1

a) Cortical NYX-2925-mediated Ca^{++} transients

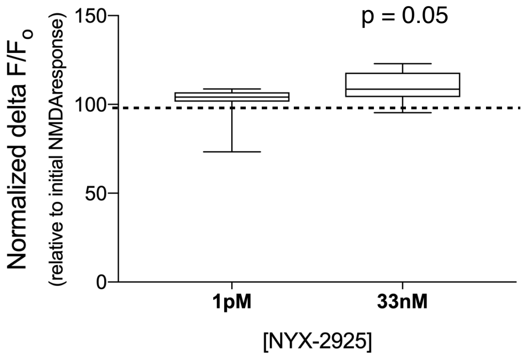

Supplement: Supplementary file 1 — Figure S1. Effects of ((2S, 3R)‐3‐hydroxy‐2‐((R)‐5‐isobutyryl‐1‐oxo‐2,5‐diazaspiro[3,4]octan‐2‐yl) butanamide on calcium transients following N‐methyl‐d‐aspartate receptor activation. Table S1. Complete ontological analysis of differentially expressed hippocampal proteins following treatment with ((2S, 3R)‐3‐hydroxy‐2‐((R)‐5‐isobutyryl‐1‐oxo‐2,5‐diazaspiro[3,4]octan‐2‐yl) butanamide (1 pM/30 min). Table S2. Complete ontological analysis of differentially expressed hippocampal proteins following treatment with ((2S, 3R)‐3‐hydroxy‐2‐((R)‐5‐isobutyryl‐1‐oxo‐2,5‐diazaspiro[3,4]octan‐2‐yl) butanamide (30 nM/30 min). Data S1. Supplementary result. [file JNC-152-523-s001.pdf]
